# Discrete magma injections drive the 2021 La Palma eruption

**DOI:** 10.1126/sciadv.adg4813

**Published:** 2023-07-05

**Authors:** Teresa Ubide, Álvaro Márquez, Eumenio Ancochea, María José Huertas, Raquel Herrera, Juan Jesús Coello-Bravo, David Sanz-Mangas, Jack Mulder, Alice MacDonald, Inés Galindo

**Affiliations:** ^1^ The University of Queensland, School of the Environment; Brisbane, Australia.; ^2^ Universidad Complutense, Área de Petrología y Geoquímica; Madrid, Spain.; ^3^ Universidad Rey Juan Carlos, ESCET, Área de Geología, Tecvolrisk Research Group; Móstoles, Madrid, Spain.; ^4^ Fundación Telesforo Bravo - Juan Coello; Puerto de la Cruz, Tenerife, Spain.; ^5^ Instituto Geológico y Minero de España (IGME-CSIC); Las Palmas de Gran Canaria, Spain.; ^6^ University of Adelaide, School of Physical Sciences; Adelaide, Australia.

## Abstract

Understanding the drivers of the onset, evolution, and end of eruptions and their impact on eruption style is critical in eruption forecasting and emergency management. The composition of erupted liquids is a key piece of the volcano puzzle, but untangling subtle melt variations remains an analytical challenge. Here, we apply rapid, high-resolution matrix geochemical analysis on samples of known eruption date spanning the entire 2021 La Palma eruption. Sr isotope signatures reveal distinct pulses of basanite melt driving the onset, restart, and evolution of the eruption. Elemental variations in matrix and microcrysts track progressive invasion, and draining, of a subcrustal crystal mush. Associated variations in lava flow rate, vent development, seismicity, and SO_2_ emission demonstrate that volcanic matrix resolves eruption patterns that could be expected in future basaltic eruptions globally.

## Teaser

Volcanic matrix geochemistry tracks pulses of magma fueling eruptive behavior during the 2021 eruption at La Palma.

## INTRODUCTION

Growing human settlement and tourism on the fertile flanks of basaltic volcanoes increases associated risks. The issue is exacerbated in regions where destructive eruptions predate human memory and perceived risk is low, as sadly exemplified in recent eruptions at Kilauea’s lower east rift in 2018 (*1*) and La Palma’s Cumbre Vieja rift in 2021 (*2*). To protect human lives and infrastructure, volcano monitoring efforts have improved markedly in the last decades (*3*). In addition to seismicity, ground deformation and gas flux tracking magma movement at depth, volcano surveillance now uses the composition of erupted products to build forecasts of potential changes in eruptive behavior (*4*, *5*). Magma composition has a direct effect on the temperature and viscosity of erupted lavas (*6*), and therefore, their mobility and hazard potential, and optimized geochemical workflows increasingly overcome the barrier of rapid analysis to inform decision-makers (*4*, *7*, *8*). Outstanding challenges include forecasting the duration and termination of eruptive activity, for which it is crucial to monitor magma source and evolution through vertically extended plumbing systems (*9*, *10*). To this end, fingerprinting the input and evolution of discrete magma batches could provide a direct, yet unresolved measure of magma supply (*11*). This is particularly relevant in ocean island basalt (OIB) systems, where source heterogeneity is commonly reflected in erupted products (*12*) and can influence magma histories (*13*).

**Fig. 1.**
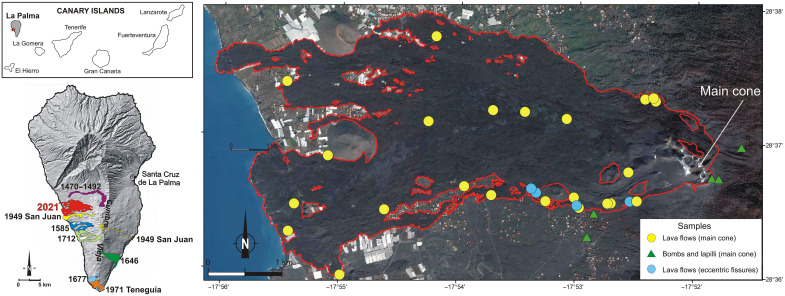
Location of the 2021 eruption at La Palma, Canary Islands, and samples analyzed in this study. The digital elevation model of La Palma shows the location of historical eruptions along the Cumbre Vieja rift. The satellite image with sample locations includes a red outline of the 2021 lava flow field from Copernicus Emergency Management Service: https://emergency.copernicus.eu/mapping/list-of-components/EMSR546. The location of the eruption on the island of La Palma is marked with a red star on the simplified map of the Canary Islands (inset).

**Fig. 2.**
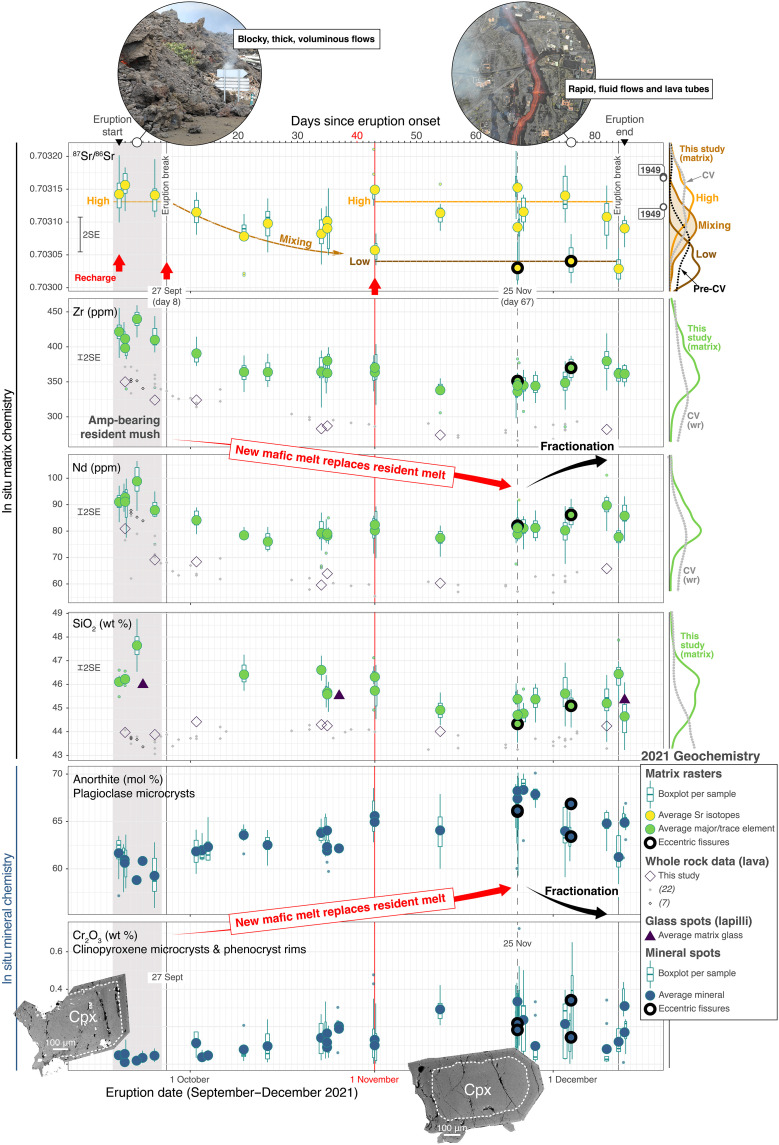
Temporal variation of matrix geochemistry and eruptive style through the 2021 La Palma eruption. Early lavas were voluminous and blocky (traffic sign for scale), with relatively evolved matrix transporting a remobilized, amphibole-bearing mush. Post eruptive break on 27 September, lavas became progressively more fluid and rapid (drone view with houses), reflecting increasing maficity (recharge magma replacing resident melt). The geochemical kink on 25 November marks a turning point from recharge to fractionation, signaling magma cooling and waning supply. Matrix analysis via laser ablation rasters (LA-MC-ICPMS for Sr isotope ratios, LA-Q-ICPMS for major and trace elements); analysis of minerals grown from erupted melts (microcrysts and rims) and lapilli glass via electron microprobe. Boxplots and averages represent ~10 measurements per sample. Error bars (left) represent uncertainty on measurements (2× SE); electron microprobe data uncertainty smaller than symbol. Red arrows mark melt chemistry milestones; note progressive variations in the first half of eruption and decoupling of elemental and isotope signatures in second half. Sr-radiogenic melts (High) follow recent eruptions at Cumbre Vieja [right; CV Kernel Density Estimate (KDE); GeoRoc; data S6], including the 1949 eruption with reported isotope heterogeneity (*39*). Sr-unradiogenic melts (Low) are similar to pre-Cumbre Vieja values [Pre-CV KDE; (*39*)]. Matrix compositions are similar to lapilli glass, whereas lava whole rocks follow similar trends but accumulate mafic minerals (table S1), also evident from Cumbre Vieja whole rock compilation (CV wr KDE, right). Clinopyroxene records mafic recharge throughout the eruption; dark rims represent crystallization from hot, mafic melt recycling previous, rounded cores (backscattered electron images from 19 September and 25 November; main cone). Phenocryst rims and microcrysts show limited compositional variation at the start of eruption and notable variation post-27 September. Increasing chromium (clinopyroxene) and anorthite (plagioclase) signal progressive mafic input (*16*, *64*), until the turning point where maficity drops due to fractional crystallization.

**Fig. 3.**
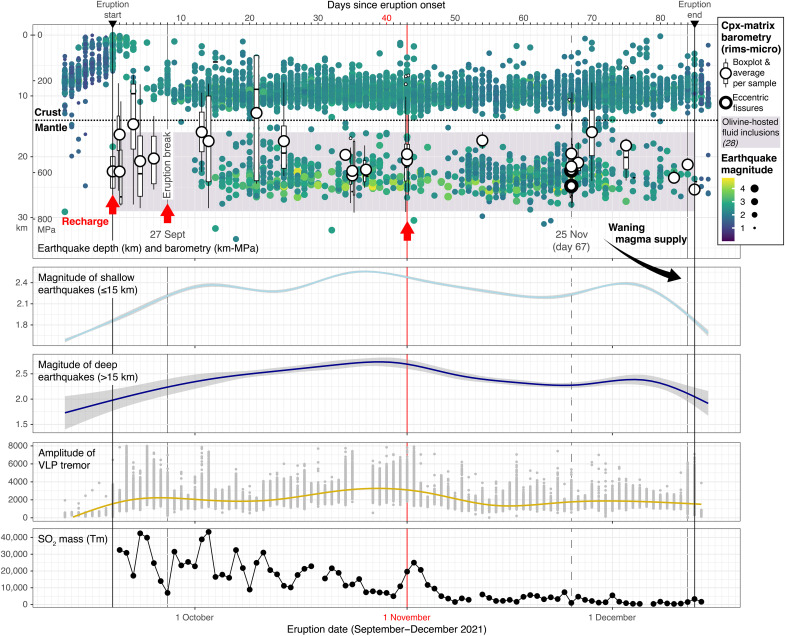
Temporal variation of seismicity, tremor, and SO_2_ emission, together with clinopyroxene-liquid barometry on phenocryst rims and microcrysts, paired with equilibrium matrix compositions. Clinopyroxene-matrix thermobarometry indicates final crystallization in upper mantle conditions throughout the eruption: 1125° ± 16°C and 529 ± 150 MPa (average ± SD, within calibration errors). Our depths are consistent with the top of the deep seismic cluster (20 to 25 km), consistent with magma accumulation (*26*), and with olivine-hosted fluid inclusion barometry, which records final magma staging before eruption [(*28*): 420 to 780 MPa]. Red arrows follow Fig. 2 milestones in melt chemistry, including the split in isotope compositions on 1 November which coincides with peaks in monitoring signs. Maxima in earthquake magnitude and VLP tremor amplitude around day 40 precede the appearance of unradiogenic Sr-Os isotope compositions (Fig. 2 and fig. S2), followed by a sharp positive peak in SO_2_ mass consistent with the arrival of undegassed magma. Decreasing seismicity and SO_2_ in early December agree with declining magma supply, as inferred from incipient fractionation of erupted products (Fig. 2). Earthquake data from INVOLCAN (*26*). VLP tremor component computed by (*17*) from INVOLCAN monitoring data. Curves for shallow and deep earthquakes and for VLP tremor are smoothed conditional means. SO_2_ daily emissions measured by TROPOMI (https://so2.gsfc.nasa.gov/). Clinopyroxene-melt barometry follows (*24*), appropriate for alkaline systems and with a calibration error of ± 170 MPa (~6 km, within the overall variation reflected by our boxplots per sample). We include average values (large circles) for samples with >1 equilibrium pair. Conversion between pressures and depths follows the crust-mantle density model based on P-wave velocity data from [(*26*); see Methods]. Depth of the crust-mantle boundary according to geophysical data offshore La Palma [14 km; (*31*)], and could be as shallow as 10 km in the region of magma intrusion feeding 2021 volcanism (*26*).

Here, we apply rapid, in situ analysis of volcanic matrix to track distinct melt pulses fueling the 19 September to 13 December 2021 eruption at Cumbre Vieja volcanic rift (La Palma, Canary Islands; Fig. 1). We resolve subtle compositional variations in erupted liquids by measuring the elemental and Sr isotope composition of volcanic matrix in samples collected systematically throughout the eruption, via laser ablation quadrupole and multicollector inductively coupled plasma mass spectrometry (LA-Q-ICPMS and LA-MC-ICPMS; Fig. 2). Our texturally constrained approach avoids mixing of whole rock geochemical signals with crystals recycled from storage regions (*14*) and represents a rapid view into the elemental and isotope composition of erupted melts avoiding very time-consuming physical separation and column chemistry (*15*). We combine matrix geochemistry with field observations, rock textures, and targeted analysis of clinopyroxene and plagioclase, ubiquitous in erupted products and characterized by slow lattice diffusion for a vast range of elements, constituting excellent proxies of melt composition. We focus on phenocryst rims and microcrysts that grew directly from erupting liquids, unlike phenocryst cores commonly crystallized from earlier intrusions (*16*). Matching clinopyroxene compositions with the liquids they crystallized from (our high-resolution matrix data), we constrain the depth of remobilization of the main subcrustal mush and compare our results with monitoring signs (Fig. 3).

The eruption was one of the most destructive of the historical record in the Canary Islands. Over 85 days of Strombolian activity with lava fountaining and ash plumes (*17*), 159 million m^3^ of lava covered >12 km^2^, destroying >1600 homes, forcing the evacuation of >7000 people and generating losses >840 million euro (*18*). Lava flows were initially thick (>10 m) and voluminous. After a short break in volcanism of a few hours on 27 September (eruption day 8), the lavas became more fluid, flowing faster though channels and tubes and producing frequent overflows and lateral outbreaks. The end of November and start of December saw two episodes of exceptionally fluid and rapid flows, generating lava “rivers” from newly opened vents thousands of meters away from the main fissure (*19*), which we term “eccentric” (Fig. 1 and fig. S1). The eruption stopped for ~1 hour on 12 December and ended the following day with a final paroxysm that emitted meter-sized bombs. The first eruptive break marked a sharp change in lava rheology (Fig. 2), linked to a mineralogical change from amphibole-clinopyroxene–bearing tephrite to clinopyroxene-olivine–rich basanite (table S1). The carrier liquids (volcanic matrix), however, formed similar microcrystalline assemblages with plagioclase, clinopyroxene, olivine, Fe-Ti oxides, and glass. Below, we explore chemical variations in the microcrystalline lava matrix with time.

## RESULTS AND DISCUSSION

### Magma recharge, mixing, and fractionation modulate eruptive activity

Our results uncover the drivers of the onset, evolution, and termination of volcanism (Fig. 2). Erupted liquids are substantially processed basanite-tephrite [matrix MgO 4.7 ± 0.7 weight % (wt %); fig. S2 and data S1], in agreement with filtering of OIB melts to eruptible ~5 wt % MgO through upper mantle reservoirs (*14*). This is different from whole rock data, partly modulated by crystal content (from 10 to 28 volume % phenocrysts, typically ~20 volume %; table S1) and with magnesium contents increasing up to >8 wt % MgO as the main phenocryst assemblage transitioned from amphibole and clinopyroxene to more abundant clinopyroxene and olivine after the eruption break on 27 September. Microcrystalline matrix compositions are similar to lapilli glass (Fig. 2 and fig. S2); however, glass measurements can only provide the composition of the bulk erupted liquid in glassy samples like fine tephra and are less versatile in lavas.

Within the general matrix homogeneity, decreasing concentrations of silica and incompatible elements (including high field strength elements like Zr and rare earth elements like Nd; Fig. 2) suggest increasing mafic invasion from the eruptive break in late September until late November. Texturally, mafic recharge is recorded in reversely zoned clinopyroxene phenocrysts throughout the eruption, with variably rounded, green, sodic ferroaugite cores with patchy zoning surrounded by MgO-rich (Mg# 69-84) titanaugite rims with common sector zoning (figs. S3 and S4). Clinopyroxene rims and microcrysts show increasing Cr_2_O_3_ concentrations from the first eruptive break until late November (Fig. 2), suggesting increasing melt maficity with time (*16*). In parallel, plagioclase microcrysts show increasing anorthite contents from labradorite to bytownite (Fig. 2). As a late-stage phase, plagioclase represents a particularly faithful proxy for the composition of erupting melt. On rare occasions, plagioclase also makes large (centimeter-size) crystals with substantially lower anorthite content (andesine) that occur as single crystals or glomerocrystic clusters and may include green clinopyroxene similar to phenocryst cores, as well as apatite and titanite (fig. S5). This is typical of phonolite assemblages in La Palma, which represent evolved, cold magmatic environments relative to basanites-tephrites (*20*, *21*). Considering their size, rarity, and occurrence mostly restricted to October products, together with their evolved mineralogy, we consider the large andesines as evolved antecrysts recycled by ascending magmas. While mineral temporal variations may not be observed without separating inherited cores from rims and microcrysts (*22*), we show mineral populations crystallized directly from the matrix melt (clinopyroxene and plagioclase rims and microcrysts) define clear variations that correlate with matrix chemistries (lasered matrix; Fig. 2). Critically, our chemical proxies for erupted liquids define a kink in late November from mafic recharge to melt fractionation, when incompatible element concentrations start to increase and the maficity of phenocryst rims and microcrysts starts to decrease (Fig. 2). According to thermal satellite data, our geochemical kink on 25 November (eruption day 67) heralds a decline in lava emission rate [from 26 November following (*17*); from 2 December following (*23*)], thus we interpret that the kink from recharge to fractionation indicates waning magma supply.

Matrix geochemical changes are limited but link clearly with the evolution of the eruption, resolving variations unseen in whole rock geochemistry (Fig. 2). We interpret: (i) the early eruption was driven by recycling of a relatively evolved, hydrous crystal mush generating thick, voluminous amphibole-bearing tephrite lavas; (ii) from the first eruptive break onward, progressively more mafic basanite liquids generated increasingly fluid and rapid lavas; until (iii) a geochemical kink from replenishment to differentiation heralded the end of the eruption, with only ~10% fractional crystallization recorded at eruption termination (fig. S6). Crucially, interactions between older, evolved, H_2_O-rich stored magma and hotter deliveries of magma are common in the initial phases of fissure eruptions, and progressive emptying of the mush can lead to increased lava fluidity and hazard potential [e.g., at Kilauea; (*4*)] because key magma properties including density and viscosity are strongly affected by magma composition (*6*). Maficity peaks have been observed with the evolution of other basaltic eruptions [e.g., 2021 paroxysms at Mount Etna; (*8*)]. If aligned with decreasing unrest and magma discharge, the switch from increasing to decreasing maficity may identify dropping magma supply and possibly eruption termination (at La Palma, just over 2 weeks after the 25 November 2021 maficity climax).

Pre- and syn-eruptive magma recharge and mixing occurred at upper mantle depths according to clinopyroxene-matrix barometry (Fig. 3). Combining our phenocryst rim and microcryst analyses with their respective matrix compositions, equilibrium clinopyroxene-liquid pairs return relatively constant temperatures and pressures of crystallization throughout the eruption [1125° ± 16°C and 529 ± 150 MPa average ± SD, within the calibration errors of ± 33°C and ± 1*7*0 MPa, (*24*); fig. S7]. Crystallization temperatures are consistent with measured lava flow temperatures [commonly >1100°C and up to 1140°C near the vent; (*25*)]. To convert crystallization pressures to depths, we use P-wave velocity measurements at increasing depth below La Palma (*26*) and the V_P_-density relationship in (*27*) to build a crust-mantle density model. Following this model, crystallization at 529 MPa equates to 20 km depth (± 6-km calibration error; Fig. 3). Our results agree with high-precision barometry in olivine-hosted fluid inclusions across the eruption, which suggest a final, main level of magma stagnation between 420 and 780 MPa (*28*). Thermobarometry on clinopyroxene-melt equilibrium pairs from previous Cumbre Vieja eruptions suggests that magma fractionation focuses mostly between 430 to 780 MPa and 1100° to 1160°C (*29*), also in agreement with our data. Clinopyroxene phenocryst rims and microcrysts from the 2021 eruption are euhedral and commonly sector-zoned, indicating dynamic crystallization environments at low degrees of undercooling, consistent with incipient magma ascent (*30*). Hence, our pressure and temperature estimates represent crystallization upon mafic invasion, mush remobilization, and the onset of magma movement toward the surface, as described for other alkaline basaltic systems such as Mount Etna (*16*, *30*).

Seismicity measured during the eruption defines two earthquake clusters [(*26*); Fig. 3]: deep seismicity in the upper mantle (20 to 25 km) and shallow seismicity at the base of the crust (10 to 12 km), with the crust-mantle boundary located at ~14 km according to geophysical data offshore La Palma (*31*) but as shallow as 10 km in the region of repeated magma intrusion feeding Cumbre Vieja volcanism (*26*). Our depths for magma remobilization and incipient ascent agree with the top of the deep seismicity, which started in October and reached higher magnitude than the shallow earthquake swarm (Fig. 3). High *V*_P_/*V*_S_ ratios at this depth range are interpreted to represent accumulation of a large magma-filled rock volume (*26*). We note that the earthquake catalog in (*32*) locates both levels of syn-eruptive seismicity comparatively deeper than (*26*) (fig. S8), with our barometry estimates falling in the intermediate aseismic zone which nevertheless is interpreted as a mushy reservoir for magma storage and eruption build-up, as recorded by repeated precursory seismicity since 2017 (*32*, *33*).

Long-term precursory seismicity may have reflected recharge of the growing and evolving mush from deeper levels in the plumbing system, priming the reservoir until a critical volume that led to eruption (*34*, *35*). Shallow seismicity started 1 week before the eruption onset and migrated rapidly to the surface, tracking the opening of the conduit and defining an unexpectedly rapid acceleration of unrest (*2*, *36*) that could be related to volatile saturation propelling magma from the crust-mantle boundary to the surface (*14*). The double-tier magma evolution, including dominant deep storage in the upper mantle and short-term storage in the lower crust, is typical of OIBs as informed by mineral and fluid inclusion barometry and has been extensively documented at La Palma as well as the neighboring island of El Hierro, hosting the most recent eruption in the Canary Islands before 2021 (*37*, *38*).

### Isotopically distinct melt pulses fuel 85 days of volcanism

Matrix Sr isotope ratios unveil discrete pulses of magma fueling the 2021 La Palma eruption. ^87^Sr/^86^Sr ratios follow incompatible trace element variations in the first half of the eruption but define a step change unlike elemental patterns in the second half of the eruption (Fig. 2). Early ^87^Sr/^86^Sr ratios are highly radiogenic (High: 0.70313 average ± 0.00003 SD) and follow the progressive increase with time defined by historical eruptions at Cumbre Vieja [(*39*); fig. S9]. After the first eruption break and until the end of October, ^87^Sr/^86^Sr ratios drop but remain within the Cumbre Vieja range, defining a progressive decrease mirroring incompatible trace element trends (Mixing: 0.70309 ± 0.00003). From the start of November halfway through the eruption, Sr isotope signatures split into two groups, one highly radiogenic like early samples and the other substantially less radiogenic (Low: 0.70304 ± 0.00002), trending toward pre-Cumbre Vieja values [(*39*); Fig. 2]. The late eccentric fissures erupted Sr-unradiogenic magmas, suggesting efficient tapping of new, isotopically distinct magma batches recharging the system at depth via eccentric feeder pathways, as observed in other basaltic volcanoes [e.g., Mount Etna; (*40*)]. Sr-unradiogenic magmas also reached the surface via the main fissure, including spills coming off the lava flow field 1500 m west of the main cone on 1 November (fig. S1). Bombs erupted at the end of the eruption have similar, low ^87^Sr/^86^Sr ratios on 12 December and higher ^87^Sr/^86^Sr ratios on the 13 December paroxysm, suggesting recycling of distinct magma batches during the final explosion.

^87^Sr/^86^Sr analyses on large, evolved plagioclase antecrysts erupted in October [andesines with ~40 mole percent (mol %) anorthite, substantially lower than 60 to 70 mol % in labradorite microcrysts in the matrix; fig. S5] afford higher precision measurements on crystals inherited from more evolved, colder mushes as described for previous eruptions at Cumbre Vieja (*20*, *21*). ^87^Sr/^86^Sr plagioclase data support Sr isotope heterogeneity within 2021 products, with ^87^Sr/^86^Sr decreasing toward the rim of an antecryst cluster hosted in a less radiogenic rock matrix (fig. S5). Sr isotope zoning unveils inheritance of large andesines from isotopically radiogenic, evolved mushes (high ^87^Sr/^86^Sr melt signature at the start of the eruption) into later, less radiogenic, mafic melts crystallizing labradorite microcrysts (mixing ^87^Sr/^86^Sr melt throughout October; Fig. 2 and fig. S5). It follows our high-resolution sampling and in situ analysis resolve fine-scale Sr isotope variations within the 2021 eruption, some of which were previously observed for the 1949 eruption, which also involved distinct eruptive phases with Sr isotope heterogeneity according to high-precision whole rock TIMS data on basanite and phonotephrite samples (*39*) (Fig. 2).

Unradiogenic Sr isotope matrix data define a new signature in Cumbre Vieja. These samples are petrographically indistinguishable from radiogenic samples (table S1) and have similar elemental signatures (Fig 2 and fig S2), including normalized rare earth element patterns (cf. rare earth element (REE) plots in fig. S10). We rule out assimilation of oceanic sediments based on the lack of correlation between ^87^Sr/^86^Sr and elemental measurements in individual matrix rasters (fig. S9). Anomalous signatures are unradiogenic (instead of radiogenic as expected from contamination with seawater or crust) and similar in elemental concentrations to other samples. The elemental composition of anomalous samples is unlike rare xenoliths described in the first part of the eruption that include white xenopumice (rhyolitic in composition and considered of sedimentary origin) and grey xenopumice (phonolitic in composition and considered magmatic) [(*22*, *25*); fig. S2], making the xenoliths unlikely assimilants.

Notably, ^87^Sr/^86^Sr variations in our matrix samples correlate closely with ^187^Os/^188^Os variations in lava whole rocks sampled by (*22*) in similar locations and dates (fig. S2 and data S2). ^187^Os/^188^Os ratios decrease and then stabilize in the first half of the eruption and become heterogeneous in the second half, with Os-unradiogenic samples coinciding with our Sr-unradiogenic samples. The Os-isotope dataset of (*22*) includes one sample that we classify as eccentric based on eruption date and location. This sample belongs to the unradiogenic group, supporting efficient tapping of new magma through eccentric pathways. Samples with anomalously low ^187^Os/^188^Os have high concentrations of elemental Os, discarding crustal contamination in the unradiogenic group. In contrast, early samples with most radiogenic Os-isotope signatures show relatively low Os elemental concentrations and could be related to crustal contamination (*22*). If so, enrichment in elemental Sr may point to assimilation of phonolite magmas similar to magmatic xenoliths of grey xenopumice, as opposed to oceanic sediments with low Sr concentration (white xenopumice) (fig. S2). However, we note that Os-isotope signatures are overall consistent with uncontaminated La Palma samples [>50–parts per trillion Os; (*41*)] and the typical radiogenic high μ (μ = ^238^U/^204^Pb) (HIMU)-type metasomatized sources of the Canary plume (*42*). Samples with low ^187^Os/^188^Os deviate slightly toward typical primitive upper mantle (*43*) suggesting unradiogenic samples record weaker source metasomatism.

Seismicity and gas monitoring support syn-eruptive mixing of distinct melt injections replenishing the feeder system throughout the eruption. In particular, the split in Sr-Os signatures from the beginning of November follows an increase in deep and shallow seismicity at the end of October and coincides with a sharp, fivefold increase in peak daily SO_2_ mass (from ~5000 Tm at the end of October to a peak of ~25,000 Tm on 2 November), breaking the eruption’s decreasing trajectory of emissions (Fig. 3). Volcanic tremor amplitude computed over the Very-Long Period (VLP) frequency band [0.4 to 0.6 Hz; (*17*)] reflects the dynamics of the deep part of the conduit feeding the eruption and reaches an eruption maximum at the start of November, further supporting the arrival of fresh, undegassed magma. In addition, the volcano produced large explosions accompanied by shock waves, which previously had only occurred immediately before the first eruption break and change in lava composition. The change in monitoring signs spans a time when thermal satellite data do not suggest a notable increase in the rate of lava emission (*17*, *23*). We therefore suggest the heterogeneity in isotope compositions, increase in earthquake magnitude, increase in VLP tremor amplitude, and SO_2_ peak at the start of November reflect the arrival of undegassed magma to the shallow plumbing system.

The constancy of incompatible trace element signatures (including similar REE patterns in melts erupted from the main cone and eccentric fissures; fig. S10) yet Sr isotope variations coupled with Os-isotope compositions from (*22*) (Fig. 2 and figs. S2 and S5) suggests cryptic variations in the melt source. Distinct magma injections could have tapped isotopically heterogeneous domains in the source, as described for the mantle below the western Canary Islands (*39*). Mantle source heterogeneity has been sampled throughout single basaltic eruptions of varied duration, including the 1943–1952 Paricutin monogenetic eruption in Mexico (*44*) and the 2021 Fagradalsfjall fissure eruption in Iceland (*11*). Distinct melt inputs have been observed to correlate with eruptive behavior on decadal time scales at Kilauea (*13*) and Mount Etna (*40*). At the 2021 Cumbre Vieja eruption in La Palma, we propose that even cryptic source variations may modulate eruptive activity during single eruptions. Sustained magma replenishment could have prolonged the 85-day 2021 eruption relative to its two to four times shorter Cumbre Vieja predecessors (24-day 1971 Teneguía and 37-day 1949 San Juan eruptions), which also showed variations in magma composition throughout the eruption (*39*, *45*, *46*).

### Plumbing system configuration

Temporal variations in the composition of erupted liquids, accessed through in situ elemental and Sr isotope analysis of volcanic matrix, identify melts with similar elemental composition but distinct isotopic signatures modulating 2021 eruptive activity. Our data suggest the start of the eruption evacuated resident Sr-radiogenic Cumbre Vieja melt in a relatively evolved, amphibole-bearing hydrous mush, which could have accumulated and fractionated over 3 years of precursory seismicity (*32*). After the first eruption break on day 8 (27 September), new Sr-unradiogenic melt with pre-Cumbre Vieja signature started to mix with the dominant resident melt, and the activation of deep seismicity may have recorded melt injections progressively recharging the main reservoir (Fig. 3) and increasing melt maficity (Fig. 2). After 40 days of activity around 1 November, seismic and gas monitoring data signaled a change in the plumbing system that may be related to recharge with undegassed magmas of end-member signatures (radiogenic and unradiogenic), erupting unmixed through the second half of the eruption. New magmas would lack substantial pre-eruptive differentiation, therefore having similar elemental composition. Increasing maficity of erupted liquids recorded progressive invasion with fresh melt pulses and peaked around day 67 (25 November), when new eccentric fissures may have effectively tapped unradiogenic melt. The geochemical kink from recharge to fractionation, although subtle, was followed by a decrease in lava emission rates (*17*, *23*), seismicity, and SO_2_ emissions consistent with waning magma supply, heralding eruption cessation 18 days later.

Mineral-melt thermobarometry on clinopyroxene phenocryst rims and microcrysts grown from the final erupted liquids (matrix) indicates crystallization at depths coincident with the deep syn-eruptive seismicity (*26*) and with high-precision fluid inclusion barometry marking the final main region of magma staging (*28*). Clinopyroxene phenocryst rims and microcrysts are sector-zoned and MgO-rich relative to phenocryst cores, implying crystallization upon recharge under conditions of magma undercooling, possibly induced by mush evacuation and incipient ascent. Therefore, our results agree with a main region of magma accumulation in the upper mantle, which could overlay deeper regions of magma stagnation generating clinopyroxene cores, as reported for previous La Palma eruptions [e.g., (*20*)]. Some of our barometry results plot close to the crust-mantle boundary, particularly at the start of the eruption, suggesting possible short-term magma storage at the base of the crust, considered the main locus of fractionation of phonolites (*21*), relatively common at La Palma (*29*, *47*). Shallow storage may enhance magma cooling and fractionation forming the amphibole-bearing mush recycled at the start of the eruption, as well as the large, evolved plagioclase antecrysts erupted mostly in October. We note, however, that recycled plagioclase antecrysts contain inclusions of green clinopyroxene, considered to crystallize at mantle depths beneath La Palma (*20*, *21*).

Subtle geochemical differences in volcanic matrix correlate with eruption rate, style, and evolution, as well as geophysical and geochemical measures of unrest (Figs. 2 and 3). Our observations can be reconciled with a model of mush remobilization followed by eruption of increasingly mafic melt until the exhaustion of fresh magma supply enabled the onset of fractional crystallization. Following the growth of a relatively evolved, hydrous mush with radiogenic Sr signatures that follow recent Cumbre Vieja eruptions, the arrival of hot magmas with contrasting Sr isotope signatures may have unlocked the mush and triggered eruption. The general homogeneity of erupted basanite liquids highlights preferential eruption of relatively fractionated mafic liquids in ocean island basalt settings (*14*). In detail, progressive invasion of the subcrustal mush led to eruption of increasingly mafic liquids generating increasingly rapid and fluidal flows with mixed to high Sr isotope signatures. At the beginning of November, geochemical and geophysical indicators suggest a possible change in the magma plumbing system configuration that could be related to collapse of brittle crustal material above the reservoir owing to magma withdrawal (*26*) and led to tapping of distinct undegassed melts. Mixing of different magma batches may have become less efficient, leading to the simultaneous eruption of end-member magmas. Liquids erupted from eccentric vents forged alternative pathways that tapped into recharge magma with low-Sr isotope signatures. The geochemical switch from replenishment to fractionation provided the first sign of cooling and differentiation of the melt, signaling eruption wind down. If the assumption that deep seismicity from 2017 marked the onset of mush development is correct, then the mush grew over moderate time scales of several years and unlocked rapidly in 2021 on time scales of days, providing a priming-to-eruption timeframe relevant to La Palma and other alkaline basaltic systems. More generally, rapid, in situ elemental and isotope geochemical analysis of volcanic matrix visualizes magma supply modulating eruption style and duration, defining chemical proxies that may assist future monitoring and eruption forecasting in active volcanoes.

## MATERIALS AND METHODS

### Samples, eruption dates, and vents

We undertook continued field sampling of freshly erupted lavas and minor tephras throughout the eruption (Fig. 1, fig. S1, and table S1), building a high-resolution sample set that includes many samples buried by later eruptive products from the eruption. Eruption dates were carefully back-calculated from sampling dates combining field observations, sample temperatures, and publicly available footage from Remotely Piloted Aircraft flights (drone companies Ticom Soluciones and Dron Services and the Instituto Geológico y Minero de España: https://info.igme.es/eventos/Erupcion-volcanica-la-palma/videos; https://youtube.com/playlist?list=PLSpgHow5v2CqJEdfeUPo5SHopvu24BOnV) as well as satellite imagery (Copernicus Emergency Management Service: https://emergency.copernicus.eu/mapping/list-of-components/EMSR546). This information was also used to assess eruptive vents (main cone versus eccentric fissures) for each sample. Samples are amphibole-bearing tephrites from 19 to 27 September, until the first eruption break, and pyroxene-olivine–rich basanites from the afternoon of 27 September until the end of the eruption. Texturally, samples are porphyritic to microporphyritic with phenocrysts dominated by amphibole-clinopyroxene in the early tephrites and clinopyroxene-olivine in the following basanites. Plagioclase dominates the microcrystalline matrix and occurs as rare, large, evolved antecrysts in October products (fig. S5). Our samples do not contain mantle or crustal xenoliths.

### Quantification of rock textures

A representative selection of samples were point-counted for modal estimates of major mineral constituents (~1000 points per thin section). Results highlight the petrological change marked by the eruptive break on 27 September, from amphibole-clinopyroxene–bearing tephrite to clinopyroxene-olivine–rich basanite (table S1).

### Whole rock geochemistry

Major and trace element concentrations were analyzed on whole rock powders of selected lava samples via lithium metaborate/tetraborate fusion, followed by rapid digestion of the resulting molten bead in weak nitric acid, and solution analysis by inductively coupled plasma optical emission spectroscopy (major elements) and inductively coupled plasma mass spectrometry (trace elements) in Actlabs, Canada (4Lithores lithogeochemistry routine: https://actlabs.com/geochemistry/lithogeochemistry-and-whole-rock-analysis/lithogeochemistry/). Quality monitor standards included BIR-1a and BCR-2, returning accuracy typically better than 1 to 3% for major elements and 5 to 15% for trace elements relative to accepted values. Results are provided in data S2, together with literature whole rock data for the 2021 eruption (*7*, *22*).

### High-resolution matrix geochemistry

We used LA-Q-ICPMS for major and trace element analysis and LA-MC-ICPMS for Sr isotope analysis (data S1). All experiments were undertaken at The University of Queensland Centre for Geoanalytical Mass Spectrometry, Radiogenic Isotope Facility (UQ RIF-lab). The laser was programmed to raster across microcrystalline matrix areas along an array of overlapping spots. Details on laser parameters, gas flows, analyzed masses, and standardization for each mass spectrometer are summarized in table S2. We measured ~10 rasters per thin section for each of two methods (fig S5).

The laser ablation system is an ASI RESOlution 193-nm excimer ultraviolet ArF laser with a dual-volume Laurin Technic ablation cell, operated with GeoStar Norris software. Ablation was performed in ultrapure He (grade 5.0, 99.999% purity) to which the Ar makeup gas and a trace amount of N_2_ were added for efficient transport and to aid ionization. For elemental analysis, we used 50-μm-diameter rasters with a repetition rate of 10 Hz, fluence of 3 Jcm^−2^, and a horizontal scanning rate of 5 μm/s. The quadrupole mass spectrometer is a Thermo iCap RQ operated with Qtegra software. The instrument was tuned with scans on NIST SRM 612 glass. We used matrix-matched BCR-2G basaltic glass as calibration standard for all major and trace elements, following (*48*). Data reduction was undertaken with iolite 4 software (*49*). To quantify major elements, we normalized the sum of major oxides to 100 wt % offline, following (*50*). Results are similar to lapilli matrix glass erupted on the same or similar date and analyzed with electron microprobe (Fig. 2 and fig. S2). To quantify trace elements, we used Si concentrations obtained in the previous step as internal standard (varying from 20.7 to 21.9 wt % Si per sample); in the few instances where the sample was not analyzed for major elements, we used data from another sample erupted on the same or similar date. Results were screened for visually unnoticed mineral cargo using Ca-Sc-Cr (clinopyroxene), Mg-Ni-Mn (olivine), Al-Sr (plagioclase), Ti-V-Cr (Fe-Ti oxides), and P-Ce (apatite). Accuracy and precision were monitored using BHVO-2G and GSD-1G matrix-matched glass reference materials as quality monitor standards (data S3; http://georem.mpch-mainz.gwdg.de/). Accuracy was typically better than 5 to 10% relative to accepted values (except for Cu, typically better than 20%). Accuracy on Si, used as internal standard to quantify trace elements, was better than 1 to 2%. Precision was typically better than 1 to 5% relative standard deviation (RSD). Limits of detection were at the sub–parts per million (ppm) level for trace elements and <2 to 4 ppm for major elements (except P < 20 ppm and Ca < 120 ppm).

Radiogenic Sr isotope measurements were conducted on a Nu Instruments Nu Plasma II multi collector inductively coupled plasma mass spectrometer (MC-ICP-MS), following the method outlined in (*51*). The mass spectrometer was operated at a radio frequency (RF) power of 1300 W, and measurements were taken over 0.2-s integration intervals on Faraday cups operating in static mode. The measured ion beams spanned masses 82 to 88, including half masses (82.5 to 75.5). The mass spectrometer was tuned in two stages. The first stage of tuning optimized signal sensitivity and the peak shape of Sr isotope masses (84 to 88) and was conducted during ablation of an in-house reference material comprising a modern mollusc shell with approximately 3000 ppm Sr. Gas flows and instrument parameters were adjusted until a signal of at least 4 V on mass 88 was achieved. The second stage of tuning was conducted during ablation of NIST SRM 610 and aimed to suppress oxide formation by reducing Th/ThO^+^ to <1% and minimized REE^2+^ interferences by ensuring that half mass peaks did not overlap Sr isotope mass peaks.

Matrix material was analyzed for Sr isotopes by rastering across the sample with an 80-μm spot diameter operating with a repetition rate of 10 Hz, fluence of 3 Jcm^−2^, and a horizontal scanning rate of 3 μm/s. A typical analysis involved 45 s of background measurement followed by approximately 120 to 180 s of ablation and 12 s of washout. The analytical session used standard-sample bracketing with primary and secondary reference materials analyzed every five unknowns. BHVO-2G was used as a primary standard for normalizing the interference-corrected ^87^Sr/^86^Sr measurements, BCR-2G was used to calibrate Rb mass bias, and MPI‐DING reference glasses T1-G, KL2-G, and StHs6/80-G were analyzed as secondary standards to assess accuracy (data S4). We applied the same analytical setup for analysis of a representative plagioclase antecryst cluster (fig. S5).

Data reduction and interference corrections were performed using iolite 4 software (*49*) following the approach detailed in (*51*), which is similar to that described in (*52*). Matrix measurements were corrected for isobaric interferences from Kr, Rb, REE^2+^, and calcium dimers or calcium-argide species (CaCa/CaAr). Plagioclase measurements were corrected for Kr, Rb, and CaCa/CaAr isobaric interferences. The isobaric ^86^Kr interference was removed by baseline subtraction of the pre-ablation, on-peak signal on mass 86. The ^87^Rb interference was removed by monitoring ^85^Rb and peak stripping using the canonical ^87^Rb/^85^Rb. The instrumental mass bias for Rb was calibrated using a bracketing standard of known ^87^Sr/^86^Sr (BCR-2G reference glass) following the approach outlined in (*53*). Doubly charged Er and Yb interferences were removed by monitoring non-interfering species on half masses (e.g., ^167^Er^2+^ on 83.5 and ^173^Yb^2+^ on 86.5) and peak stripping using canonical isotope ratios. Interferences from calcium dimers and calcium argides on masses 84 to 88 were removed by peak stripping using the measured signals on masses 82 and 83 and canonical Ca and Ar isotope ratios. The measured signals on masses 82, 83, and all half-masses during analyses of unknowns were typically low (<0.1 mV), suggesting that the interferences due to REE^2+^ and CaCa/CaArs are small.

Our matrix-melt proxies retrieve the composition of magmatic liquids without recycled crystals, defining subtle variations with time (Fig. 2) and providing input melt compositions for mineral-melt thermobarometry (Fig. 3) and for modeling melt physical properties and their implications for flow mechanisms. The method is rapid and versatile, as it can be applied to volcanic matrix with glassy to microcrystalline texture. Therefore, our analytical approach can be applied to a wide range of eruptions, including purely effusive activity with limited emission of glassy tephra, as well as tephra collected throughout eruptions.

### Rayleigh fractional crystallization

We modeled fractional crystallization of trace elements starting from a relatively unevolved lava matrix erupted on 26 November from the main cone (IG2611L2), using the average phenocryst assemblage in the basanites erupted after the first eruptive break (64%cpx + 28%ol + 2%mag + 6%pl) and mineral/melt partition coefficients in alkali basalts (*54*). The subtle decrease in compatible metals (e.g., Ni) and increase in incompatible elements (e.g., Zr, Th, Rb, and La) in matrix erupted from late November to mid- December (eruption cease) require very limited fractional crystallization (~10%; fig. S6).

### Mineral chemistry

Electron microprobe spot analysis targeted minerals ubiquitous in erupted products and crystallized from the erupting melt: plagioclase and clinopyroxene microcrysts (<100-μm crystal width), as well as clinopyroxene phenocryst rims, avoiding the very outermost rims related to rapid growth at high cooling rates upon final magma ascent and eruption (*16*). We used a JEOL JXA-8900M electron microprobe equipped with four wavelength dispersive spectrometers, hosted at the Centro Nacional de Microscopía Electrónica of the Universidad Complutense de Madrid (Spain). Analyses were performed using an accelerating voltage of 15 kV and an electron beam diameter of 5 μm, with a current of 20 nA to maximize precision of clinopyroxene data and barometry estimates (*55*). In addition, we analyzed lapilli matrix glass for comparison with matrix major element data, using a low beam current of 7 nA and a defocused beam of 10 μm to minimize Na loss. Elemental counting times for all analyses were 10 s on the peak and 5 s on each of two background positions. Corrections for inter-elemental effects used a ZAF (Z, atomic number; A, absorption; F, fluorescence) procedure. Calibration was undertaken on mineral standards from the Smithsonian Institution of Washington. Analytical precision was 0.5 to 6% for major elements and better than 10% for oxides with concentrations of <1.5 wt %. Specifically, analyses on clinopyroxene phenocryst rims and microcrysts, used for thermobarometry estimates, had major element precision of 0.4% (Si) to 4.8% (Na). Results are provided in data S5.

### Clinopyroxene-melt thermobarometry

To constrain the temperature and depth of magma recharge and mixing, we applied clinopyroxene-liquid calibrations appropriate to alkali magma composition at La Palma [(*24*); calibration errors ± 33°C and ± 170 MPa] on clinopyroxene phenocryst rims and microcrysts, paired with equilibrium matrix compositions. This approach focuses on final crystallization from erupted liquids, analyzed with our high-resolution approach avoiding crystal accumulation on whole rock compositions (*14*). The targeted clinopyroxene populations are euhedral and commonly sector-zoned, indicating dynamic crystallization conditions at low degrees of undercooling (*30*), consistent with crystallization during early magma remobilization and ascent. Following (*30*), we used both prism and hourglass sector compositions in clinopyroxene phenocryst rims and microcrysts, provided they were in equilibrium with the matrix. We used the average matrix composition of the sample hosting each crystal as putative equilibrium liquid; where unavailable, we used the average matrix composition of a sample erupted on the same or similar date. We tested for clinopyroxene-melt equilibrium (i) applying the Fe-Mg exchange coefficient [*K*_D_(Fe-Mg)^cpx-melt^ = 0.28 ± 0.08; (*56*)] and (ii) comparing diopside + hedenbergite components measured in clinopyroxene and those predicted from the liquid composition [∆DiHd within 10%; (*57*)]. We only used clinopyroxene-matrix pairs that passed both equilibrium tests (*n* = 211), obtaining consistent temperature and pressure results throughout the eruption: 1125° ± 16°C and 529 ± 150 MPa (average ± SD, within the calibration error of the thermometer and barometer; fig. S7). Clinopyroxene-melt equilibrium pairs and thermobarometry results [computed with the open-source ThermoBar Python3 package (*58*)] are provided in data S5.

To assess different thermobarometers and the effect of water content on pressure and temperature (PT) estimates, we coupled the same barometer (*24*) with the thermometer of [(*56*); equation 33 with calibration error ± 45°C]. This thermometer considers the water content of the melt, and solved iteratively with the barometer of (*24*), returns water-dependent PT estimates, relevant to western Canary Island basanites as they are volatile-rich (*59*–*61*). This thermobarometry combination is also commonly applied to mafic alkaline magmas (*62*). We considered the same clinopyroxene-putative equilibrium initial pairs and equilibrium filters as above, together with a range of melt water contents appropriate for undegassed Canary magmas [1 to 3 wt % H_2_O; (*59*–*61*)]. Increasing water contents from 1 to 3 wt % H_2_O, we obtained decreasing temperatures from 1129° to 1097° ± 18°C and pressures from 540 to 450 ± 150 MPa [20 to 17 ± 6 km depth using our density model based on seismic data in (*26*)]. This test (fig. S7) shows variations with water content and between PT approaches are minor and within the calibration error of the thermobarometers, supporting our original estimates for final crystallization of clinopyroxene from matrix melts upon ascent through the upper mantle (Fig. 3 and data S5).

We also tested thermobarometry results following recent machine learning approaches (*63*) and obtained similar temperatures of 1129° ± 9°C (average ± SD) but lower pressures of 148 ± 64 MPa, equivalent to 6 ± 2 km (fig. S7). We consider our original estimates following (*24*) most likely to reflect crystallization conditions because: (i) machine learning approaches lack the thermodynamic basis of traditional thermobarometry (*24*, *56*); (ii) model uncertainties are larger and encompass the magnitude of pressure results; (iii) equivalent depths are shallower than the storage depth constrained by high-precision fluid inclusion barometry (*28*) and deep seismicity (*26*) for the 2021 eruption. Nevertheless, all pressure estimates overlap within the uncertainty of calibrations and agree with crystallization of clinopyroxene rims and microcrysts upon ascent imposing undercooling and hence sector zoning (*30*).

### Seismicity and conversion of pressures to depths of crystallization

Short-term pre-eruptive seismicity and syn-eruptive seismicity were sourced from Instituto Volcanológico de Canarias (INVOLCAN) (*26*) and Instituto Geográfico Nacional (IGN) (*32*) monitoring catalogs.

To calculate the crust-mantle density profile beneath the volcano, we used P-wave velocity data measured at La Palma in (*26*) from the surface down to 30 km depth, transformed into density values following equation 1 from (*25*). We obtained a depth-pressure linear correlation of depth (km) = 0.0378 pressure (MPa), with a regression coefficient of *R*^2^ = 0.9997. Applying this equation to our clinopyroxene-liquid barometry results, we obtained crystallization depths of 20 ± 6 km (average ± SD; Fig. 3 and data S5). These values indicate upper mantle crystallization throughout the eruption, coincident with the top of the deep syn-eruptive earthquake cluster measured by (*26*) (20 to 25 km; Fig. 3). This region represents a low P-wave and S-wave velocity zone with high *V*_P_/*V*_S_ ratio, consistent with magma accumulation (*26*). Our final clinopyroxene crystallization depths also agree with high-precision olivine-hosted fluid inclusion barometry recording the main melt storage reservoir [420 to 780 MPa, (*28*); 16 to 29 km using our density model as shown in Fig. 3, similar to 15 to 27 km calculated assuming densities of 2.8 kg/m^3^ for 14 km of crust and 3.1 kg/m^3^ for the upper mantle with presence of melt; (*28*)]. We propose clinopyroxene rims and microcrysts crystallized upon mafic recharge, mush remobilization, and ascent, imposing conditions of undercooling and dynamic crystallization conducive to the development of sector zoning (*30*).

We tested the pressure-depth conversion following regional data by IGN [(*32*); comparison with data in (*26*) is presented in fig. S8]. Clinopyroxene-melt depths converted using P-wave velocity data by (*32*) down to 24 km depth also indicate final clinopyroxene crystallization at upper mantle depths throughout the eruption. Such depths fall between the deep and shallow seismic clusters measured during the eruption, at the top of their interpreted aseismic main mush reservoir (*32*). Regardless of the seismic model used, our data indicate clinopyroxene phenocryst rims and microcrysts formed upon ascent from the main mush reservoir. Following the detailed assessment of both seismic models by (*28*), we favor the dataset by (*26*), who also provide a local crust-mantle velocity model instead of the regional model by (*32*). Therefore, we use data by (*26*) through the text and in Fig. 3.

### Gas geochemistry monitoring data

Syn-eruptive sulfur dioxide flux was sourced from TROPOMI Volcanic SO_2_ satellite measurements (Copernicus Sentinel-5 Precursor Orbiter; https://so2.gsfc.nasa.gov/).

## Supplementary Materials

### This PDF file includes:

Supplementary Text Figs. S1 to S10 Legends for table S1 and S2 Legends for data S1 to S6 References
