# Supplementary material for: Discrete magma injections drive the 2021 La Palma eruption
Source: Sci Adv. 2023 Jul 5;9(27):eadg4813. doi: 10.1126/sciadv.adg4813 (PMC10321733; doi:10.1126/sciadv.adg4813)
Supplement: Supplementary file 1 — Supplementary Text Figs. S1 to S10 Legends for table S1 and S2 Legends for data S1 to S6 References [file sciadv.adg4813_sm.pdf]

Science Advances
AAAS

Supplementary Materials for

Discrete magma injections drive the 2021 La Palma eruption

Teresa Ubide *et al.*

Corresponding author: Teresa Ubide, t.ubide@uq.edu.au

Sci. Adv. **9**, eadg4813 (2023)
DOI: 10.1126/sciadv.adg4813

The PDF file includes:

Supplementary Text
Figs. S1 to S10
Legends for table S1 and S2
Legends for data S1 to S6
References

Other Supplementary Material for this manuscript includes the following:

Tables S1 and S2
Data S1 to S6

Supplementary Materials

Supplementary Text: Eruption history of the 2021 La Palma eruption

The 2021 La Palma fissure eruption took place on the western side of the Cumbre Vieja rift, affecting populations, farmland, and infrastructure (Fig. 1). The eruption started on 19th September 2021 with amphibole-bearing tephrite lavas and tephras emitted along a main NW-SE eruptive fissure with multiple vents, soon developing into a single large cone which partially collapsed in a few days (*65*). The beginning of the eruption produced fast aa-type lava flows and one thick, blocky lava flow with high emission rates (around 50 m^3/s, (*66,67*) Fig. 2), as well as high SO_2 emissions, lapilli and ash columns and explosions with associated shock waves. Magma emission and seismic tremor abruptly stopped for a few hours in the early morning of 27th September. Activity resumed in the late afternoon with lava fountains and fast lava flows of pyroxene-olivine-rich basanite, which persisted until the end of the eruption (Fig. 2). Deep seismicity from ~30-40 km began a few days after the restart of activity and continued until the end of the eruption (Fig. 3). Until early November, erupted products included felsic xenopumices similar to those erupted at Teneguía volcano in 1971 and during the 2011 submarine eruption on El Hierro island (*68,69*).

After the eruption break on 27th September, volcanic activity was mainly characterized by Strombolian explosions and lava fountaining from multiple vents, with advancing and branching aa-type lava flows and occasional pahoehoe flows. Thick blocky flows were not produced after the break. The lavas often flowed over previous flows and through channels and tubes, and formed two lava deltas when reaching the sea (Fig. 1, fig. S1). Satellite data on SO_2 atmospheric emission (Fig. 3) and thermal flux reflecting lava emission rate *(17,23)* decreased gradually after the break suggesting declining magma output, with punctuated episodes of increased activity, including around 1st November. Repeated episodes of overflow of previous lava flow levees, cone reorganization and vent opening led to the development of several flow fronts that gradually widened the lava field. On 25th November and 4th December, two remarkable episodes of opening of new vents in eccentric locations up to ~2,000 m from the main cone, associated with the appearance of fractures and fissures (*19*), emitted purely effusive, fast, and short-lived

pahoehoe-type lava flows. Eruptive activity stopped for ~1h on 12th December, resuming with a 6,000-m high eruptive column and the fall of meter-size bombs. Finally, in the afternoon of 13th December, a final explosive paroxysm produced the highest eruptive column of the eruption (8,500 m) and further bombs. After the paroxysm, the eruptive activity and seismic tremor stopped, marking the end of the eruption on 13th December 2021.

Supplementary Figures, Tables and Data:

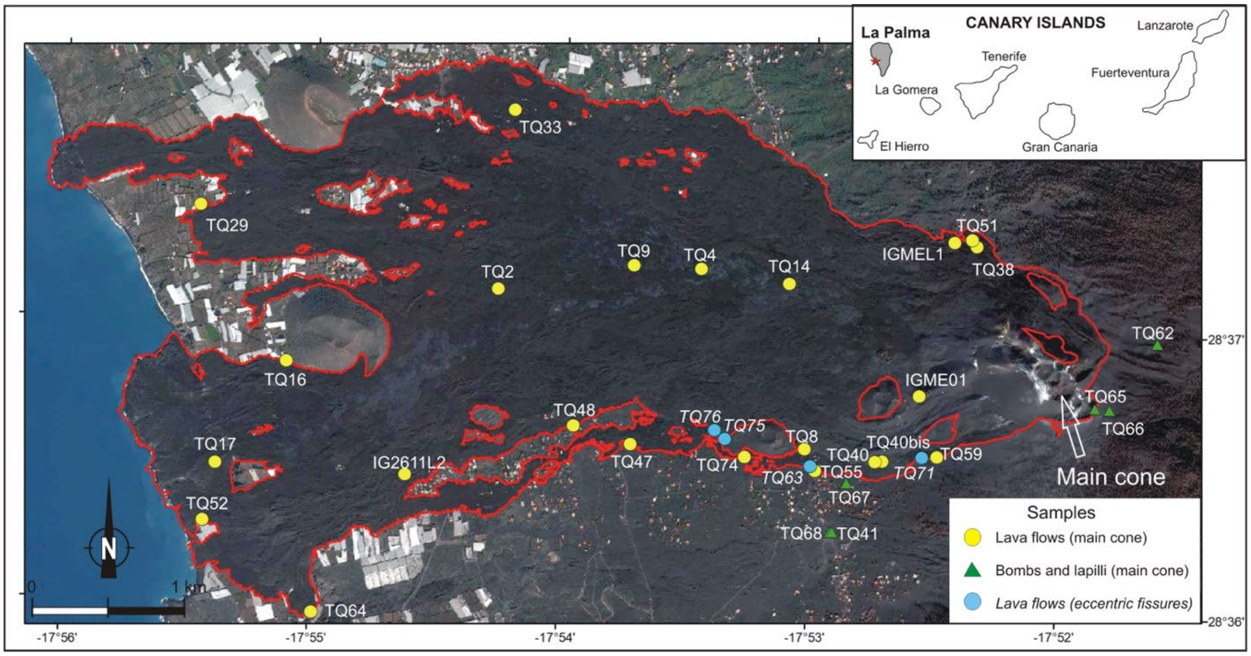

Fig. S1. Satellite image with outline of the 2021 lava flow field (red; from COPERNICUS Emegency Management Service: https://emergency.copernicus.eu/mapping/list-of-components/EMSR546) and locations of analyzed samples, including sample names. The location of the eruption on the island of La Palma is marked with a red star on the simplified map of the Canary Islands (inset), and together with other historical eruptions in Figure 1.

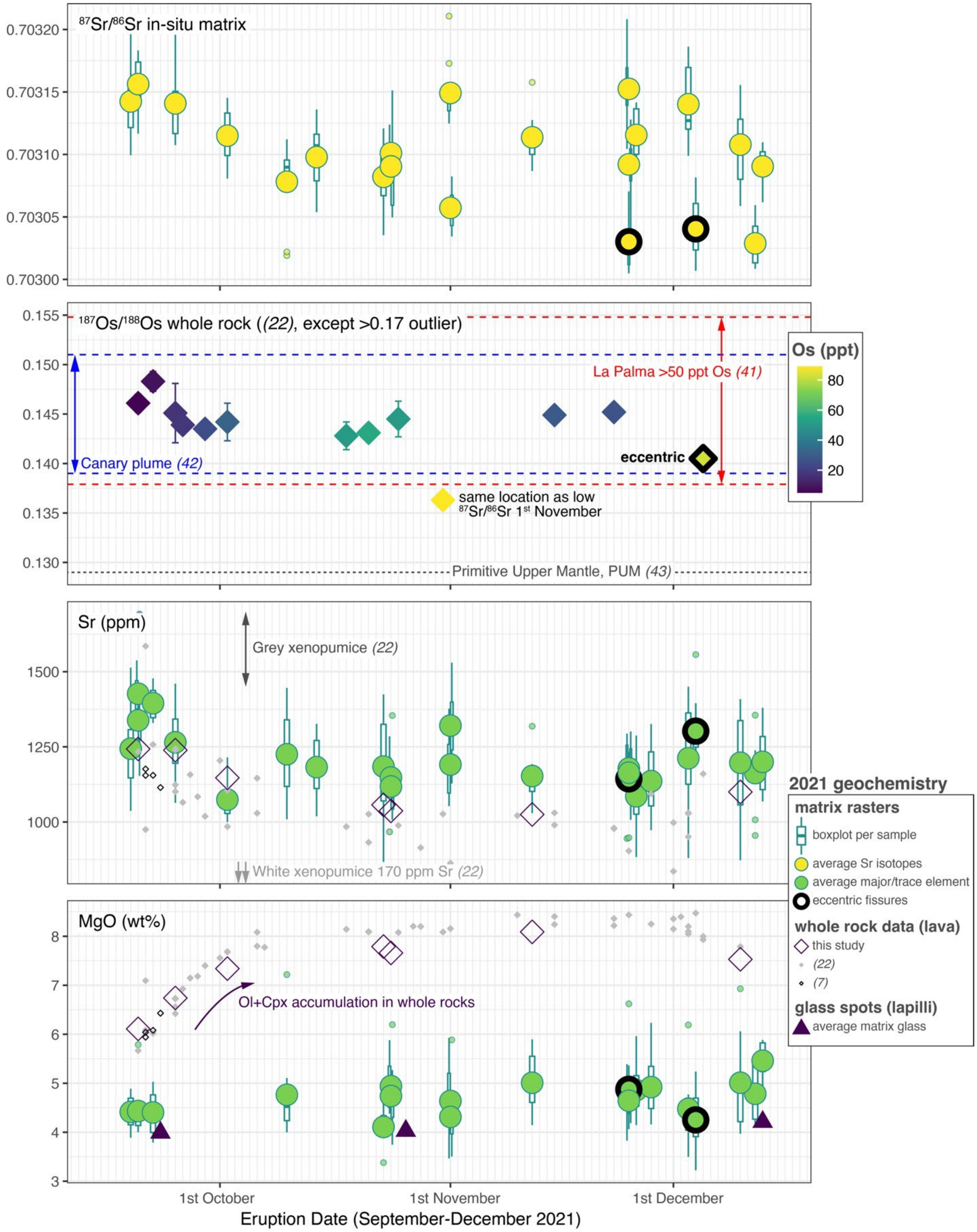

Fig. S2. Assessment of $^{87}Sr/^{86}Sr$ variations through the 2021 eruption at La Palma, comparing our matrix in-situ $^{87}Sr/^{86}Sr$ (as in Fig. 2), Sr (ppm) and MgO (wt%) with whole rock and glass compositions, including whole rock $^{187}Os/^{188}Os$ and Os (ppt; color scale) by *(22)* (excluding one outlier with >0.17 $^{187}Os/^{188}Os$ and <50 ppt Os, as typical of samples related to crustal

contamination). $^{87}Sr/^{86}Sr$ matrix data mirror $^{187}Os/^{188}Os$ whole rock variations, including decreasing signatures at the start of the eruption and a split in isotope signatures in the second half of the eruption, with unradiogenic values corresponding to samples from equivalent locations and eruption dates, including those from eccentric vents (note a one-day offset between the eruption dates of our Sr-unradiogenic samples and the Os-unradiogenic samples from *(22),* within sampling error; table S1, data S2). Overall, $^{187}Os/^{188}Os$ variations fall within the typical range of La Palma unaltered samples (>50 ppt Os; *(41)*) and the typical signature of the Canary plume, defined on non-contaminated samples with Os concentrations between 12 and 232 ppt *(42)* and reflecting HIMU-type metasomatized sources (e.g., *(41,42)*). This suggests minimal contamination of 2021 melts, with constant MgO compositions in matrix (note whole rocks are affected by accumulation of pyroxene and particularly olivine, common after the first eruption break at the end of September; table S1) yet variable isotope compositions suggesting subtle changes in the magma source. The Sr-Os-unradiogenic samples, including those from eccentric vents, have Os-isotope signatures trending towards the modern primitive upper mantle *(43),* suggesting a subtly less metasomatized source. The early, more radiogenic melts agree with typical Cumbre Vieja Sr-isotope signatures (Fig. 2), and we interpret the linear trend between radiogenic and unradiogenic signatures from late September to mid-October as mixing between end-member signatures through the first half of the eruption. Despite their lower elemental Os concentrations, radiogenic samples are unlikely related to assimilation of oceanic crust because they have relatively high Sr concentrations, whereas white xenopumice of sedimentary origin erupted in the first part of the eruption (bulk rhyolite composition; *(25)*) have Sr concentrations one order of magnitude lower *(22).* In contrast, grey xeno-pumice fragments of phonolitic composition, also noted in earlier La Palma eruptions *(25)* have high Sr concentrations *(22)* and may suggest early 2021 melts were mixed with phonolite compositions fractionated in shallow portions of the plumbing system *(21)* before the start of the eruption. Regardless, elemental and isotope relationships suggest mixing of melts with slightly different sources throughout the 2021 eruption.

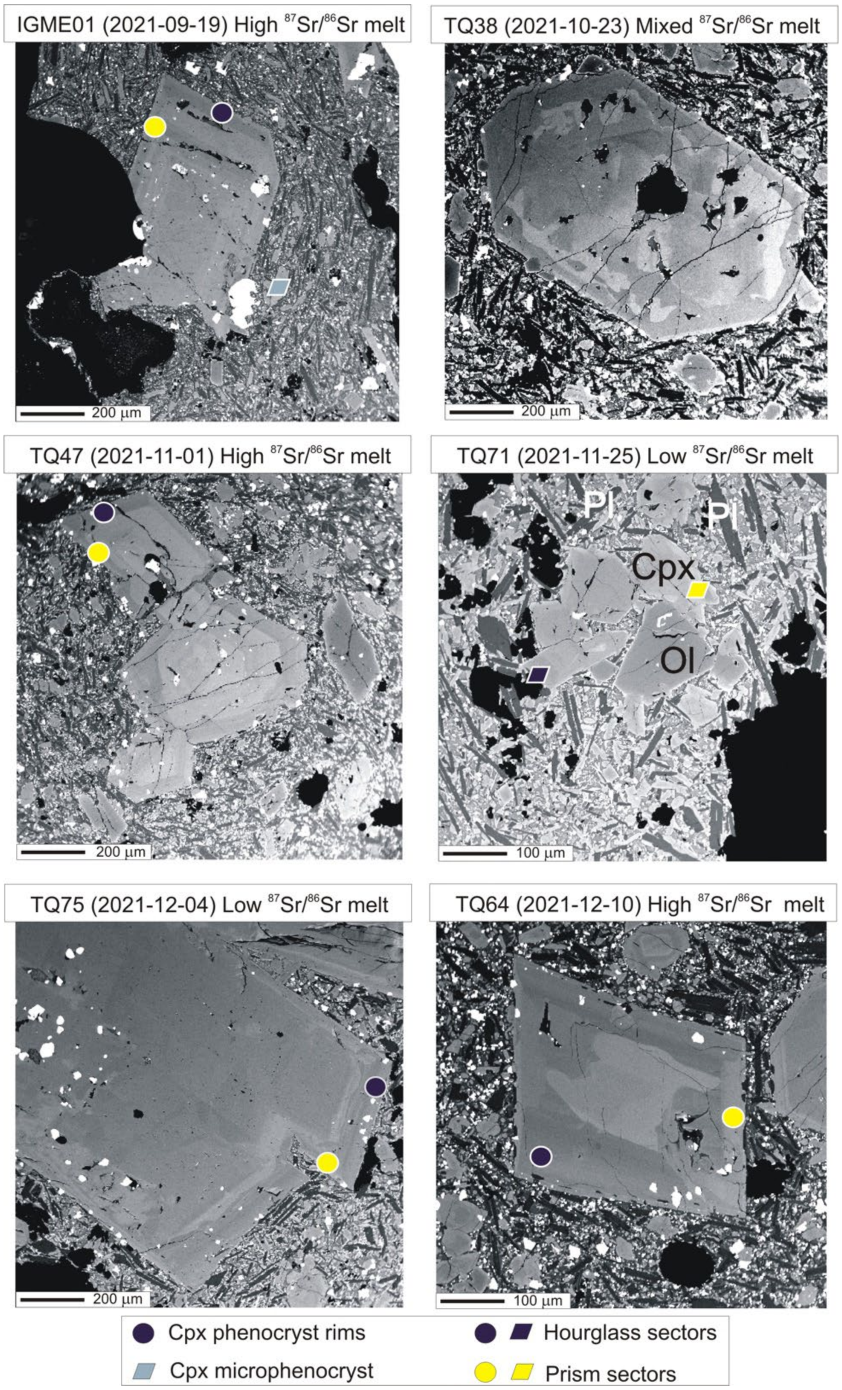

Fig. S3. Backscattered electron (BSE) images of clinopyroxene throughout the 2021 La Palma eruption, obtained via electron microprobe. Clinopyroxene phenocrysts show reverse zoning, defined by partly resorbed, patchy-zoned cores overgrown by Mg-rich rims with dark BSE contrast, which are often sector-zoned. Matrix microcrysts have similar composition and zoning to phenocryst rims. Our electron microprobe analyses and clinopyroxene-matrix thermobarometry focus on clinopyroxene phenocryst rims (circles; excepting the outermost rims with light-BSE contrast) and microcrysts (diamonds; <100 µm width), on both prism and hourglass sectors where observable. Symbols mark the location of electron microprobe spot analyses, with fill colors following those in Fig. S4. The middle-right image zooms into matrix with olivine (Ol), clinopyroxene (Cpx) and plagioclase (Pl) microcrysts.

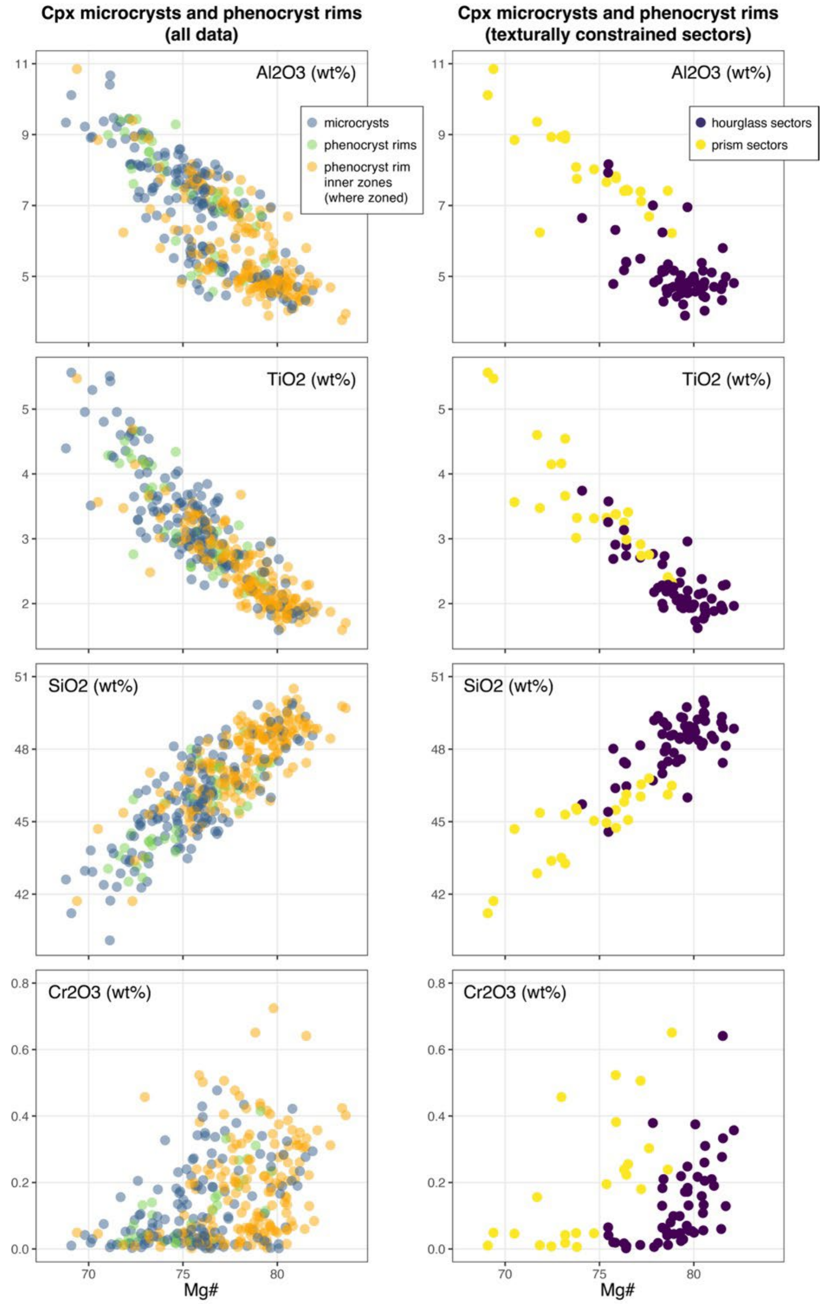

Fig. S4. Matrix clinopyroxene compositions for the 2021 La Palma eruption, in bivariate plots of major element oxides (wt%) vs. Mg#, used as maficity index (Mg# = 100 Mg / (Mg + Fe_t), where concentrations are expressed on a molar basis, and Fe_t is total iron as Fe^{2+}). The left panels include all data from clinopyroxene microcrysts and phenocryst rims (excluding outermost zones where the rims are zoned), defining a single evolutionary trend consistent with final crystallization from the carrier (matrix) melt. Clinopyroxene microcrysts and phenocryst rims are often sector zoned, and the right panels show only analyses where the sector (hourglass or prism) could be identified following the morphological model for titanoaugite *(30)* (cf. example images in fig. S3). Sector zoning explains much of the variability in Al_2O_3, TiO_2, SiO_2 and Mg#, indicating dynamic crystallization conditions under a mild undercooling regime, such as during magma ascent *(30)*. Cr_2O_3 concentrations are less dependent on sector zoning and reach maxima in inner phenocryst rims recording mafic recharge *(16)*.

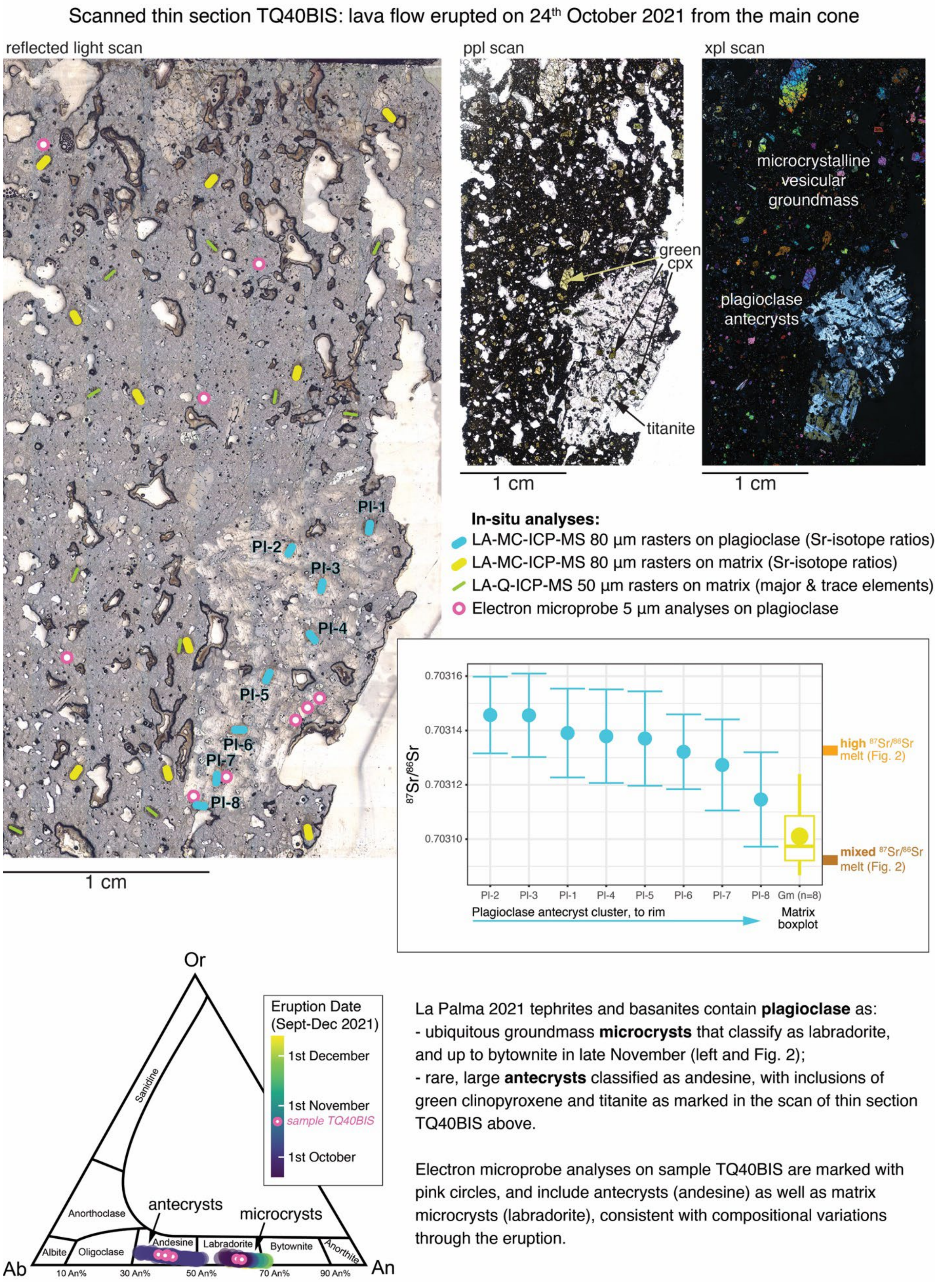

Fig. S5. $^{87}Sr/^{86}Sr$ and elemental variations across plagioclase antecrysts, microcrysts, and the rock matrix, together with an illustration of our in-situ matrix analysis methodology for $^{87}Sr/^{86}Sr$

and major+trace elements. Top images are microscope scans of a La Palma 2021 basanite thin section under reflected and transmitted light (ppl: plane polarized light; xpl: cross polarized light). The reflected light scan provides the location of individual plagioclase and matrix LA-ICPMS rasters, as well as electron microprobe measurements on plagioclase. The sample contains one of the rare, large plagioclase antecryst clusters erupted in October 2021, with inclusions of green clinopyroxene similar to phenocryst cores observed through the eruption, as well as titanite, typical of La Palma phonolites *(20,21)*. Plagioclase antecrysts are evolved andesine, low in anorthite (An) relative to matrix microcrysts (which typically classify as labradorite, and have compositions up to bytownite in samples erupted at the end of November 2021; see also Fig. 2). Plagioclase antecrysts in this sample have more radiogenic $^{87}Sr/^{86}Sr$ signatures than rims and host matrix, indicating recycling of large, low-An plagioclase crystallized from evolved, radiogenic melts (high $^{87}Sr/^{86}Sr$ signature; Fig. 2) into later, more primitive and less radiogenic melts (mixing $^{87}Sr/^{86}Sr$ signature; Fig. 2).

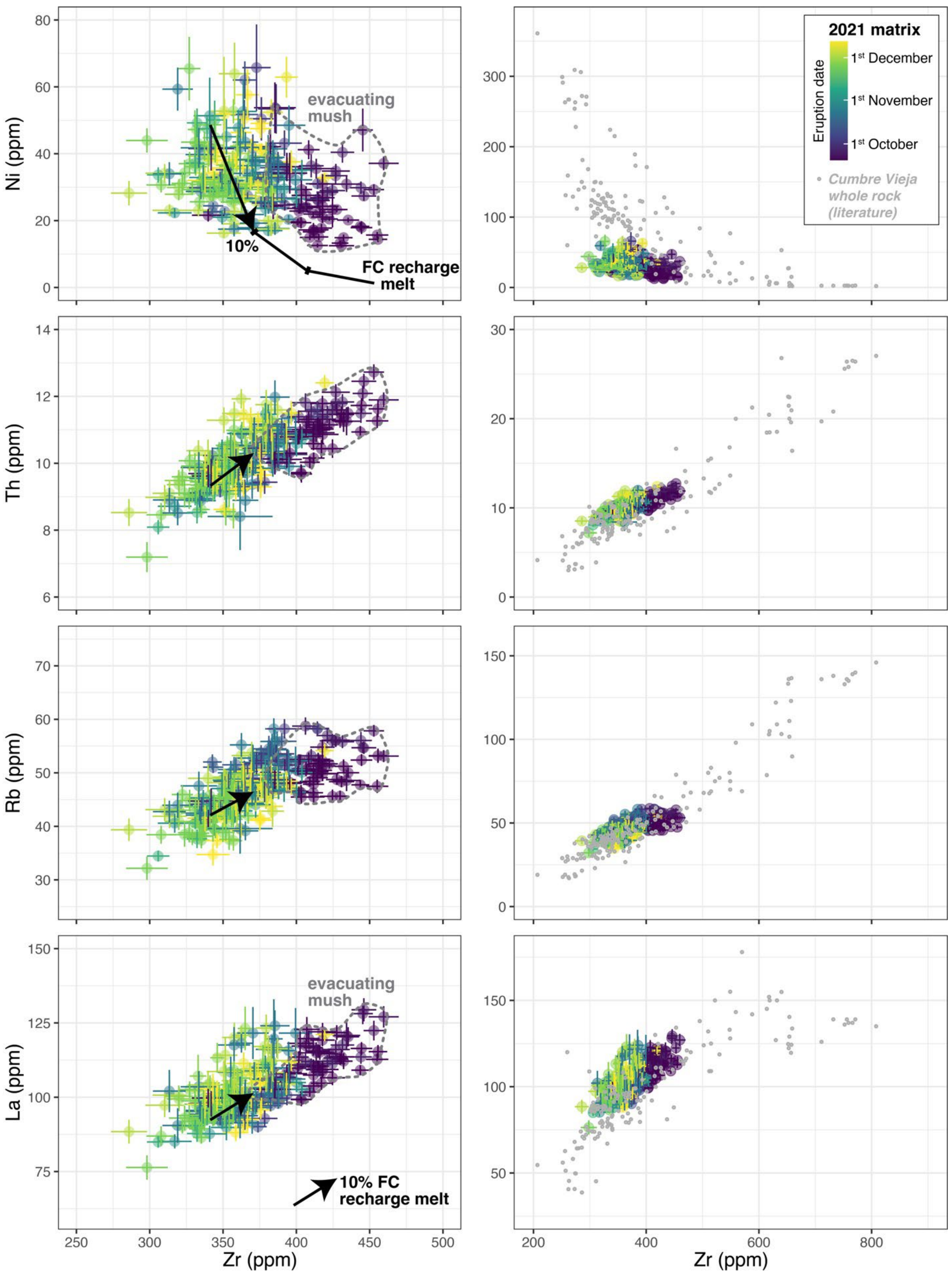

Fig. S6. Trace element composition of microcrystalline matrix throughout the 2021 La Palma eruption, together with model lines for Rayleight fractional crystallization of recharge melts.

Symbols represent individual matrix rasters, color-coded with eruption date and with error bars representing analytical uncertainty (2x standard error). Matrix erupted before the 27th September eruption break is distinctly more evolved (purple symbols have higher contents in high field strength elements, light ion lithophiles and rare earth elements, including Zr, Th, Rb and La; together with slightly lower concentrations in compatible metals like Ni) and represents remobilization and mixing with a hydrous (amphibole-bearing) tephrite mush. Toward the end of the eruption in mid-December, the mild decrease in compatible metals and increase in incompatible trace elements defined by green to yellow symbols agrees with ~10% fractional crystallization of liquids erupted at the subtle maficity climax in late November (Fig. 2). Fractional crystallization modeling considers the phenocryst assemblage observed in erupted basanites (dominated by clinopyroxene and olivine; table S1) and mineral/melt partition coefficients in alkali basalt *(54)*. The right panels show our matrix data broadly agrees with trends defined by whole rock literature data from Cumbre Vieja, with higher concentrations in incompatible elements for evolved samples (compiled and curated from GeoRoc: Geochemistry of Rocks of the Oceans and Continents, http://georoc.mpch-mainz.gwdg.de/georoc/; data S6). Some whole rock literature data are enriched in compatible metals (e.g., Ni) and depleted in incompatible elements (e.g., Zr, Th, Rb, La) due to accumulation of mafic minerals *(14)*. In contrast, our matrix data represent crystal-free melts that are relatively processed and constant in composition.

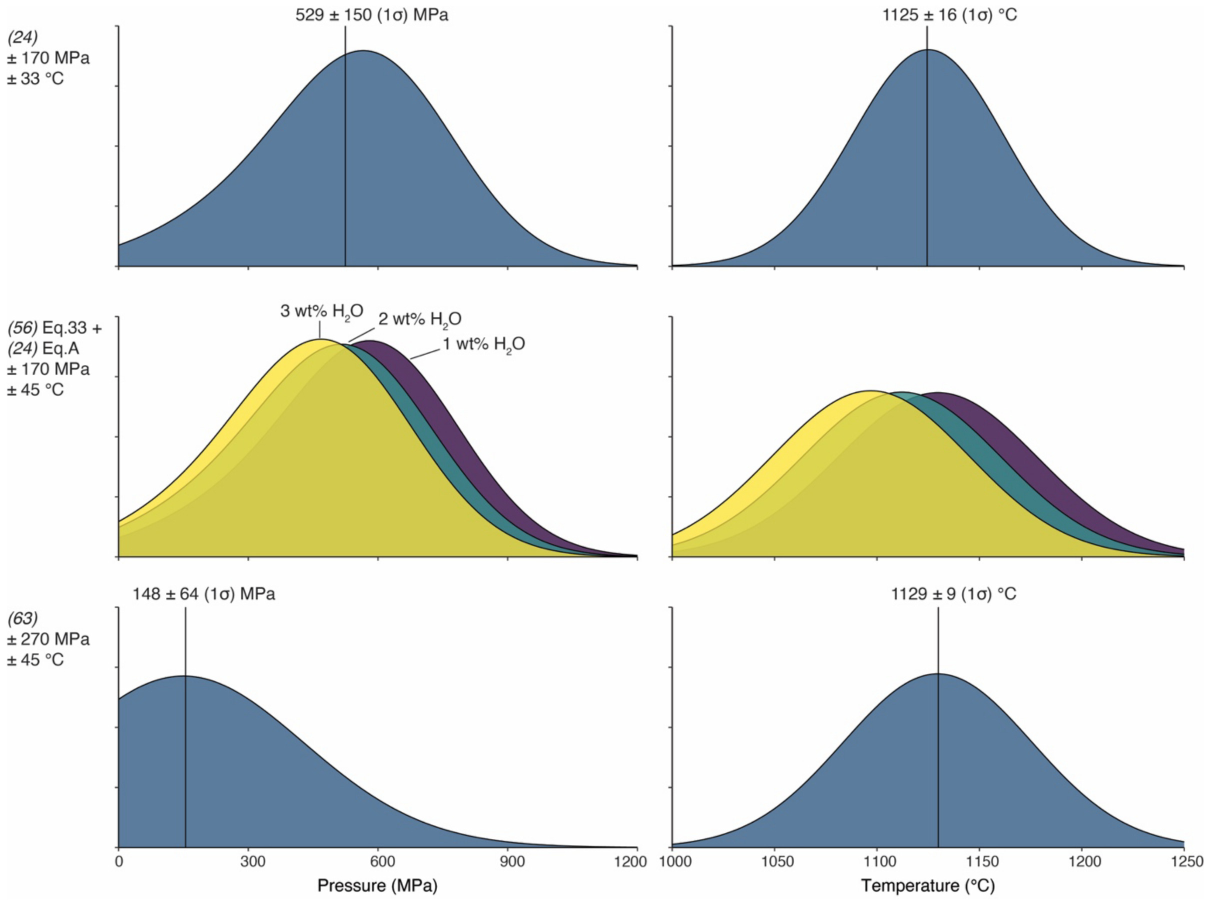

Fig. S7. Kernel density estimates of clinopyroxene-liquid thermobarometry results (pressure left; temperature right) following thermobarometric calibrations appropriate for alkaline magmas (top-preferred: *(24);* middle: *(56)* water-dependent thermometer with *(24)* barometer) and recent machine learning approaches (bottom: *(63)*). Bin width represents model uncertainty. For the water-dependent thermobarometry approach, we consider melt water contents appropriate for undegassed Canary magmas: 1, 2 and 3 wt% H_2O *(59,60,61).* Within this water range, results from traditional calibrations based on thermodynamic principles are in good agreement with each other (top and middle). Machine-learning (bottom) returns similar temperatures but lower pressures, which nevertheless agree with crystallization upon ascent yet with larger uncertainties. Pressure results from the top panel are transformed to crystallization depths for plotting in Fig. 3 and fig. S8 (see crust-mantle density model in Methods).

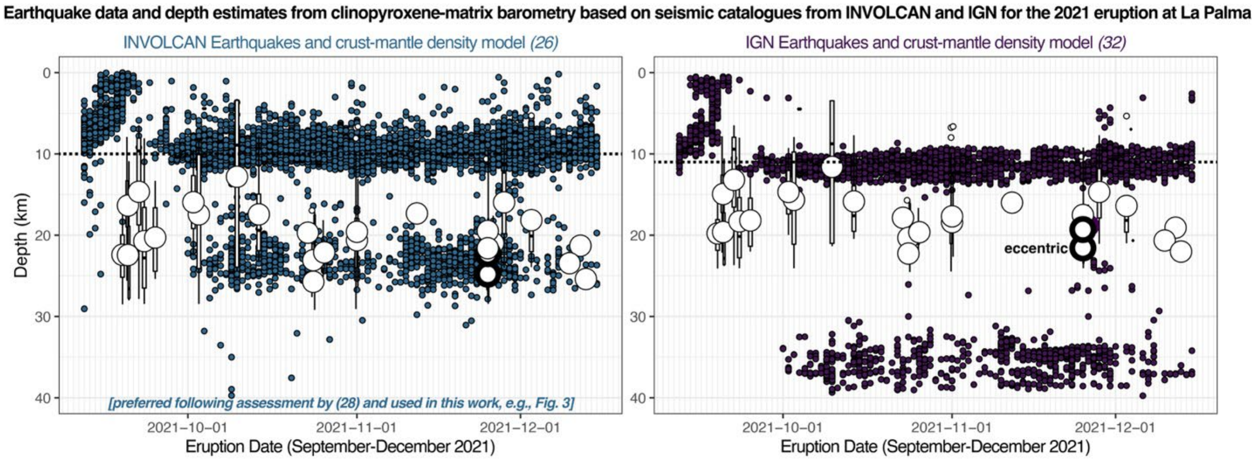

Fig. S8. Comparison of syn-eruptive earthquake catalogs from INVOLCAN *(26)* and IGN *(32)*, together with crystallization pressures of clinopyroxene phenocryst rims and microcrysts converted to depths following the crust-mantle velocity model from each study. Crust-mantle boundary (dotted line) located at 10 km *(26)* or 11 km *(32)* in the region of magma intrusion feeding 2021 volcanism. Following the assessment of both seismic models by *(28)*, we favor the dataset by *(26),* who also provide a local crust-mantle velocity model, in contrast with the regional model by *(32)*.

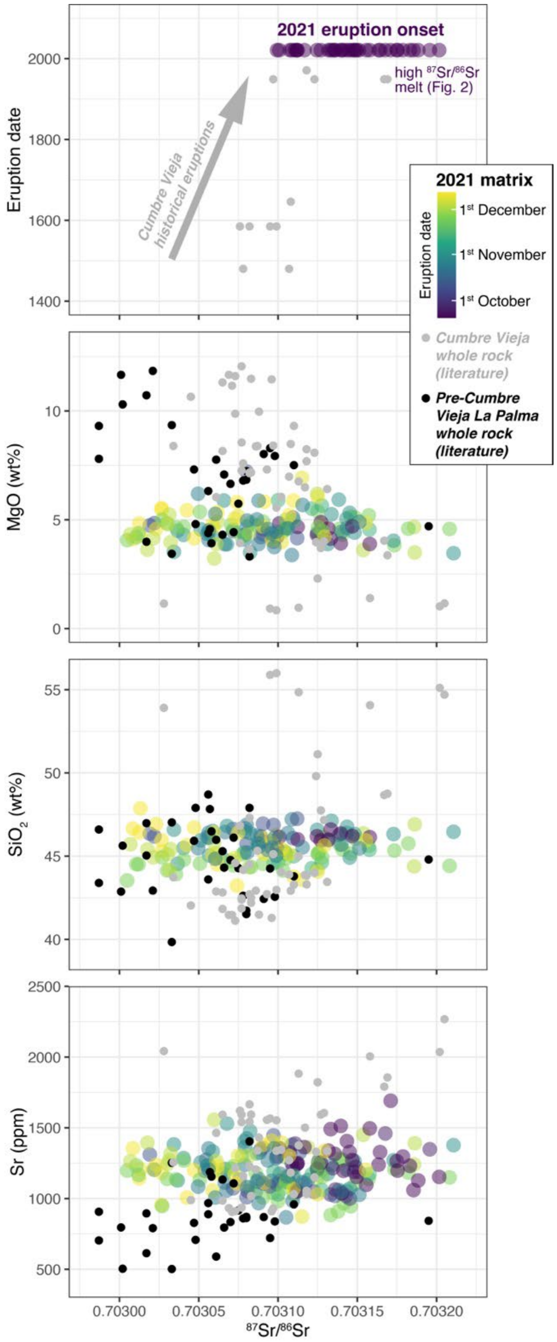

Fig. S9. $^{87}Sr/^{86}Sr$ ratios in 2021 La Palma matrix do not correlate with elemental MgO, SiO_2 or Sr, suggesting Sr-radiogenic signatures are not related to crustal contamination. This is consistent with upper mantle storage, with fast magma ascent informed by shallow pre-eruptive seismicity, and with the lack of xenoliths in our samples. Matrix data broadly agree with trends defined by La Palma whole rock literature data. Interestingly, $^{87}Sr/^{86}Sr$ ratios in the early 2021 matrix (until the first eruptive break on 27th September; top panel) follow a trend of increasing $^{87}Sr/^{86}Sr$ defined by historical eruptions at Cumbre Vieja. In contrast, some $^{87}Sr/^{86}Sr$ signatures from November and December, including those from eccentric vents, are less radiogenic, trending toward pre-Cumbre Vieja values. Colored symbols represent individual matrix rasters (this study), color-coded with eruption date. Whole rock Cumbre Vieja data compiled and curated from GeoRoc (data S6), and pre-Cumbre Vieja values from (*39*).

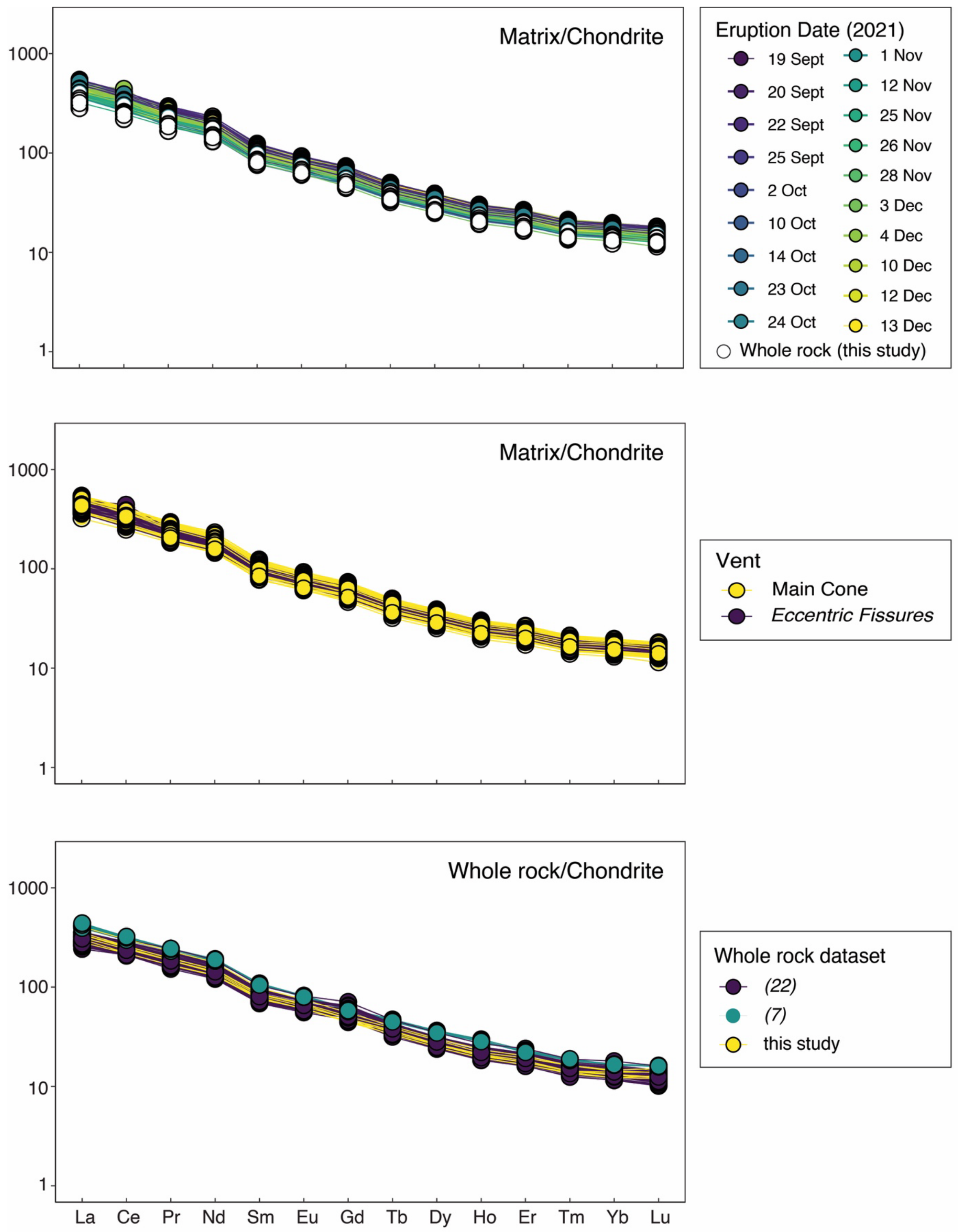

Fig. S10. Rare earth element patterns of 2021 La Palma matrix and whole rocks, normalized to chondrite *(70)*. REE patterns are similar across eruption dates, vents, and for matrix and whole rocks, suggesting a similar mantle source across the eruption. Subtle differences in isotope compositions (Fig. 2, fig. S2) unveil isotopically distinct melt batches variably mixed and tapped through time.

Table S1. Studied samples from the 2021 eruption at Cumbre Vieja rift, La Palma, Canary Islands. Modal fractions were estimated by point counting ~1000 locations per thin section. Matrix analyses were undertaken via laser ablation quadrupole and multi-collector mass spectrometry. Mineral chemistry includes clinopyroxene phenocryst rims and microcrysts, plagioclase microcrysts and antecrysts, and glass.

Table S2. Instrument parameters for matrix analysis by laser ablation quadrupole mass spectrometry (LA-Q-ICPMS) for major and trace elements, and laser ablation multi-collector mass spectrometry (LA-MC-ICPMS) for Sr-isotopes. Plagioclase Sr-isotope LA-MC-ICPMS analysis followed the same procedures, with distinct interference corrections as detailed.

Data S1. Geochemistry of volcanic matrix for the 2021 eruption at Cumbre Vieja rift, La Palma, from this study. Major and trace element data analyzed via LA-Q-ICPMS rasters (elemental concentrations in ppm; major element oxides in wt.%). Sr-isotope data analyzed via LA-MC-ICPMS rasters.

Data S2. Whole rock geochemistry for the 2021 eruption at Cumbre Vieja rift, La Palma, from this study, *(7),* and *(22).* Major elements in wt% oxides; trace elements in ppm unless otherwise specified (low abundance highly siderophile elements Os, Ir, Ru, Pt, Pd, Re; *(22)*); LOI loss on ignition. Data from this study were analyzed by solution ICP-OES for major elements and ICP-MS for trace elements.

Data S3. Elemental concentrations on reference materials (RM), analyzed together with unknowns via LA-Q-ICP-MS (major elements in wt% oxides; trace elements in ppm).

Data S4. A) Sr-isotope results on reference materials (RM), analyzed together with unknowns via LA-MC-ICP-MS. B) Summary of Sr-isotope results on reference materials (RM). Recommended values are from GeoReM (http://georem.mpch-mainz.gwdg.de/).

Data S5. Electron microprobe major element data on clinopyroxene phenocryst rims and microcrysts, plagioclase microcrysts and antecrysts, and volcanic glass (wt% oxides). Clinopyroxene data are paired with putative equilibrium liquids (matrix compositions) and tested for equilibrium on Fe-Mg exchange *(56)* and DiHd components *(57).* Only clinopyroxene-matrix pairs that pass both equilibrium tests are used to obtain thermobarometric estimates, following calibrations appropriate for alkaline magmas *(24)*. Crystallization depths follow the crust-mantle density profile obtained from measurements on the speed of P-wave anomalies from the surface down to 30 km depth at La Palma, using data collected by *(26).*

Data S6. Literature compilation of geochemical data from eruptions prior to 2021 at Cumbre Vieja, downloaded and curated from GeoRoc: Geochemistry of Rocks of the Oceans and Continents, http://georoc.mpch-mainz.gwdg.de/georoc/ (major oxides wt%; trace elements ppm).

REFERENCES AND NOTES

1. B. F. Houghton, W. A. Cockshell, C. E. Gregg, B. H. Walker, K. Kim, C. M. Tisdale, E. Yamashita, Land, lava, and disaster create a social dilemma after the 2018 eruption of Kīlauea volcano. *Nat. Commun.* **12**, 1223–1223 (2021).

2. M.-A. Longpre, Reactivation of Cumbre Vieja volcano. *Science* **374**, 1197–1198 (2021).

3. M. P. Poland, K. R. Anderson, Partly cloudy with a chance of lava flows: Forecasting volcanic eruptions in the twenty-first century. *J. Geophys. Res. Solid Earth* **125**, 1 (2020).

4. C. Gansecki, R. L. Lee, T. Shea, S. P. Lundblad, K. Hon, C. Parcheta, The tangled tale of Kīlauea's 2018 eruption as told by geochemical monitoring. *Science* **366**, eaaz0147 (2019).

5. G. Re, R. A. Corsaro, C. D'Oriano, M. Pompilio, Petrological monitoring of active volcanoes: A review of existing procedures to achieve best practices and operative protocols during eruptions. *J. Volcanol. Geotherm. Res.* **419**, 107365 (2021).

6. D. Giordano, J. K. Russell, D. B. Dingwell, Viscosity of magmatic liquids: A model. *Earth Planet. Sci. Lett.* **271**, 123–134 (2008).

7. M. J. Pankhurst, J. H. Scarrow, O. A. Barbee, J. Hickey, B. C. Coldwell, G. K. Rollinson, J. A. Rodríguez-Losada, A. Martín Lorenzo, F. Rodríguez, W. Hernández, D. Calvo Fernández, P. A. Hernández, N. M. Pérez, Rapid response petrology for the opening eruptive phase of the 2021 Cumbre Vieja eruption, La Palma, Canary Islands. *Volcanica* **5**, 1–10 (2022).

8. R. A. Corsaro, L. Miraglia, Near Real-Time Petrologic Monitoring on Volcanic Glass to Infer Magmatic Processes During the February–April 2021 Paroxysms of the South-East Crater, Etna. *Front. Earth Sci.* **10**, 828026 (2022).

9. J. Kauahikaua, M. Mangan, C. Heliker, T. Mattox, A quantitative look at the demise of a basaltic vent: The death of Kupaianaha, Kilauea Volcano, Hawai'i, *Bull. Volcanol.* **57**, 641–648 (1996).

10. K. V. Cashman, R. S. J. Sparks, J. D. Blundy, Vertically extensive and unstable magmatic

systems: A unified view of igneous processes. *Science* **355**, eaag3055 (2017).

11. S. A. Halldórsson, E. W. Marshall, A. Caracciolo, S. Matthews, E. Bali, M. B. Rasmussen, E. Ranta, J. G. Robin, G. H. Guðfinnsson, O. Sigmarsson, J. Maclennan, M. G. Jackson, M. J. Whitehouse, H. Jeon, Q. H. A. van der Meer, G. K. Mibei, M. H. Kalliokoski, M. M. Repczynska, R. H. Rúnarsdóttir, G. Sigurðsson, M. A. Pfeffer, S. W. Scott, R. Kjartansdóttir, B. I. Kleine, C. Oppenheimer, A. Aiuppa, E. Ilyinskaya, M. Bitetto, G. Giudice, A. Stefánsson, Rapid shifting of a deep magmatic source at Fagradalsfjall volcano, Iceland, *Nature* **609,** 529–534 (2022).

12. A. Stracke, A. W. Hofmann, S. R. Hart, FOZO, HIMU, and the rest of the mantle zoo. *Geochem. Geophys. Geosystems* **6**, Q05007 (2005).

13. A. J. Pietruszka, D. E. Heaton, J. P. Marske, M. O. Garcia, Two magma bodies beneath the summit of Kīlauea Volcano unveiled by isotopically distinct melt deliveries from the mantle. *Earth Planet. Sci. Lett.* **413**, 90–100 (2015).

14. T. Ubide, P. Larrea, L. Becerril, C. Gale, Volcanic plumbing filters on ocean-island basalt geochemistry. *Geology* **50,** 26–31 (2022).

15. R. Magee, T. Ubide, J. Caulfield, Days to weeks of syn-eruptive magma interaction; High-resolution geochemistry of the 2002-03 branched eruption at Mount Etna. *Earth Planet. Sci. Lett.* **565**, 116904 (2021).

16. T. Ubide, B. S. Kamber, Volcanic crystals as time capsules of eruption history. *Nat. Commun.* **9**, 326 (2018).

17. C. Bonadonna, M. Pistolesi, S. Biass, M. Voloschina, J. Romero, D. Coppola, A. Folch, L. D’Auria, A. Martin-Lorenzo, L. Dominguez, C. Pastore, M. P. Reyes Hardy, F. Rodríguez, (2022). Physical characterization of long-lasting hybrid eruptions: The 2021 Tajogaite eruption of Cumbre Vieja (La Palma, Canary Islands). *J. Geophys. Res. Solid Earth* **127**, 10.1029/2022jb025302 (2022).

18. Government report by Comisión mixta para la reconstrucción, recuperación y apoyo a la isla

de La Palma, integrada por el Gobierno De España, el Gobierno De Canarias, el Cabildo De La Palma y los ayuntamientos de Los Llanos De Aridane, El Paso y Tazacorte, Informe sobre las actuaciones y medidas emprendidas tras la erupción del volcán de Cumbre Vieja (La Palma), seis meses después del inicio de la emergencia. June 2022: www.mpr.gob.es/prencom/notas/Documents/2022/060622-informe_palma.pdf

19. P. J. González, Volcano-tectonic control of Cumbre Vieja. *Science* **375**, 1348–1349 (2022).

20. A. K. Barker, V. R. Troll, J. C. Carracedo, P. A. Nicholls, The magma plumbing system for the 1971 Teneguía eruption on La Palma, Canary Islands. *Contrib. Mineral. Petrol.* **170**, 54 (2015).

21. A. Klügel, E. Albers, T. H. Hansteen, Mantle and crustal xenoliths in a tephriphonolite From La Palma (Canary Islands): Implications for phonolite formation at oceanic island volcanoes. *Front. Earth Sci.* **10,** 761902 (2022).

22. J. M. D. Day, V. R. Troll, M. Aulinas, F. M. Deegan, H. Geiger, J. C. Carracedo, G. G. Pinto, F. J. Pérez-Torrado, Mantle source characteristics and magmatic processes during the 2021 La Palma eruption. *Earth Planet. Sci. Lett.* **597**, 117793 (2022).

23. S. Plank, A. V. Shevchenko, P. d'Angelo, V. Gstaiger, P. J. González, S. Cesca, S. Martinis, T. R. Walter, Combining thermal, tri-stereo optical and bi-static InSAR satellite imagery for lava volume estimates: The 2021 Cumbre Vieja eruption, La Palma. *Sci. Rep.* **13**, 2057 (2023).

24. K. D. Putirka, H. Mikaelian, F. Ryerson, H. Shaw, New clinopyroxene-liquid thermobarometers for mafic, evolved, and volatile-bearing lava compositions, with applications to lavas from Tibet and the Snake River Plain, Idaho. *Am. Mineral.* **88**, 1542–1554 (2003).

25. J. C. Carracedo, V. R. Troll, J. M. D. Day, H. Geiger, M. Aulinas, V. Soler, F. M. Deegan, F. J. Perez-Torrado, G. Gisbert, E. Gazel, A. Rodriguez-Gonzalez, H. Albert, The 2021 eruption of the Cumbre Vieja volcanic ridge on La Palma, Canary Islands. *Geol. Today* **38**, 94–107

(2022).

26. L. D’Auria, I. Koulakov, J. Prudencio, I. Cabrera-Pérez, J. M. Ibáñez, J. Barrancos, R. García-Hernández, D. Martínez van Dorth, G. D. Padilla, M. Przeor, V. Ortega, P. Hernández, N. M. Peréz, Rapid magma ascent beneath La Palma revealed by seismic tomography. *Sci. Rep.* **12**, 17654 (2022).

27. T. M. Brocher, Empirical relations between Elastic Wavespeeds and Density in the Earth's Crust. *Bull. Seismol. Soc. America.* **95**, 2081–2092 (2005).

28. K. Dayton, E. Gazel, P. Wieser, V. R. Troll, J. C. Carracedo, H. La Madrid, D. C. Roman, J. Ward, M. Aulinas, H. Geiger, F. M. Deegan, G. Gisbert, F. J. Perez-Torrado, Deep magma storage during the 2021 La Palma eruption. *Sci. Adv.* **9**, eade7641 (2023).

29. A. Klügel, T. H. Hansteen, K. Galipp, Magma Storage and Underplating beneath Cumbre Vieja Volcano, La Palma (Canary Islands). *Earth Planet. Sci. Lett.* **236**, 211–226 (2005).

30. T. Ubide, S. Mollo, J. X. Zhao, M. Nazzari, P. Scarlato, Sector-zoned clinopyroxene as a recorder of magma history, eruption triggers, and ascent rates. *Geochim. Cosmochim. Acta* **251**, 265–283, (2019).

31. C. R. Ranero, M. Torne, E. Banda, Gravity and multichannel seismic reflection constraints on the lithospheric structure of the Canary Swell. *Marine Geophys. Res.* **17**, 519–534 (1995).

32. C. del Fresno, S. Cesca, A. Klügel, I. D. Cerdeña, E. A. Díaz-Suárez, T. Dahm, L. García-Cañada, S. Meletlidis, C. Milkereit, C. Valenzuela-Malebrán, R. López-Díaz, C. López, Magmatic plumbing and dynamic evolution of the 2021 La Palma eruption. *Nature Commun.***14**, 358 (2023).

33. J. Fernandez, J. Escayo, Z. Hu, A. G. Camacho, S. V. Samsonov, J. F. Prieto, K. F. Tiampo, M. Palano, J. J. Mallorquí, E. Ancochea, Detection of volcanic unrest onset in La Palma, Canary Islands, evolution and implications. *Sci. Rep.* **11**, 2540 (2021).

34. R. Magee, T. Ubide, M. Kahl, The lead-up to Mount Etna’s most destructive historic eruption

(1669). Cryptic recharge recorded in clinopyroxene. *J. Petrol.* **61**, egaa025 (2020).

35. M. Townsend, C. Huber, A critical magma chamber size for volcanic eruptions. *Geology* **48**, 431–435 (2020).

36. L. Passarelli, E. E. Brodsky, The correlation between run-up and repose times of volcanic eruptions. *Geophys. J. Int.* **188,** 1025–1045 (2012).

37. M.-A. Longpré, A. Klügel, A. Diehl, J. Stix, Mixing in mantle magma reservoirs prior to and during the 2011-2012 eruption at El Hierro, Canary Islands, *Geology* **42**, 315–318 (2014).

38. A. Klügel, M. A. Longpré, L. García-Cañada, J. Stix, Deep intrusions, lateral magma transport and related uplift at ocean island volcanoes. *Earth Planet. Sci. Lett.* **431**, 140–149 (2015).

39. A. Klügel, K. Galipp, K. Hoernle, F. Hauff, S. Groom, Geochemical and volcanological evolution of La Palma, Canary Islands. *J. Petrol.* **58**, 1227–1248 (2017).

40. R. Clocchiatti, M. Condomines, N. Guenot, J.-C. Tanguy, Magma changes at Mount Etna; The 2001 and 2002-2003 eruptions. *Earth Planet. Sci. Lett.* **226**, 397–414 (2004).

41. J. M. D. Day, D. G. Pearson, C. G. Macpherson, D. Lowry, J. C. Carracedo, Evidence for distinct proportions of subducted oceanic crust and lithosphere in HIMU-type mantle beneath El Hierro and La Palma, Canary Islands. *Geochim. Cosmochim. Acta* **74**, 6565–6589 (2010).

42. E. Widom, K. A. Hoernle, S. B. Shirey, H.-U. Schmincke, Os Isotope Systematics in the Canary Islands and Madeira: Lithospheric Contamination and Mantle Plume Signatures. *J. Petrol.* **40**, 279–296 (1999).

43. T. Meisel, R. J. Wlker, J. W. Morgan, The osmium isotopic composition of the Earth's primitive upper mantle. *Nature* **383**, 517–520 (1996.).

44. P. Larrea, E. Widom, C. Siebe, S. Salinas, D. Kuentz, A re-interpretation of the petrogenesis of Paricutin volcano: Distinguishing crustal contamination from mantle heterogeneity.

Chem.Geol. **504**, 66–82 (2019).

45. E. Ibarrola, Temporal modification of the basaltic materials from 1971 eruption of the Teneguia volcano. *Estud. Geol.* **Teneguía**, 15-18 (1974).

46. A. Klügel, H.-U. Schmincke, J. D. L. White, K. A. Hoernle, Chronology and volcanology of the 1949 multi-vent rift-zone eruption on La Palma (Canary Islands). *J. Volcanol. Geotherm. Res.* **94**, 267–282 (1999).

47. J. C. Carracedo, E. Rodríguez Badiola, H. Gouillou, H. J. De La Nuez, F. J. Pérez Torrado, Geology and volcanology of La Palma and El Hierro, Western Canaries. *Estud. Geol.* **57**, 175–273 (2001).

48. F. E. Jenner, H. St. C. O'Neill, Major and trace analysis of basaltic glasses by laser-ablation ICP-MS. *Geochem. Geophys. Geosystems* **13**, 10.1029/2011GC003890 (2012).

49. C. Paton, J. D. Woodhead, J. C. Hellstrom, J. M. Hergt, A. Greig, R. Maas, Improved laser ablation U-Pb zircon geochronology through robust downhole fractionation correction. *Geochem. Geophys. Geosyst.* **11**, 1–36 (2010).

50. J. Chang, A. Audétat, Petrogenesis and metal content of hornblende-rich xenoliths from two Laramide-age magma systems in southwestern USA: Insights into the metal budget of arc magmas. *J. Petrol.* **59**, 1869–1898 (2018).

51. J. Mulder, G. Hagen-Peter, T. Ubide, R. Andreasen, E. Kooijman, M. Kielman-Schmitt, Y.-X. Feng, B. Paul, A. Karlsson, C. Tegner, C. Lesher, F. Costa, New reference materials, analytical procedures and data reduction strategies for Sr isotope measurements in geological materials by LA-MC-ICP-MS. *Geostand. Geoanal. Res.* **47**, 311–336 (2023).

52. G. Hagen-Peter, C. Tegner, C. E. Lesher, Strontium isotope systematics for plagioclase of the Skaergaard intrusion (East Greenland): A window to crustal assimilation, differentiation, and magma dynamics. *Geology* **47**, 313–316 (2019).

53. M. G. Jackson, S. R. Hart, Strontium isotopes in melt inclusions from Samoan basalts:

Implications for heterogeneity in the Samoan plume. *Earth Planet. Sci. Lett.* **245**, 260–277 (2006).

54. B. Villemant, H. Jaffrezic, J.-L. Joron, M. Treuil, Distribution coefficients of major and trace elements; Fractional crystallization in the alkali basalt series of Chaîne des Puys (Massif Central, France). *Geochim. Cosmochim. Acta* **45**, 1997–2016 (1981).

55. P. E. Wieser, A. J. R. Kent, C. B. Till, J. Donovan, D. A. Neave, D. L. Blatter, M. J. Krawczynski, Barometers behaving badly: Assessing the influence of analytical and experimental uncertainty on clinopyroxene thermobarometry calculations at crustal conditions. *J. Petrol.* **64**, egac126 (2022).

56. K. D. Putirka, Thermometers and barometers for volcanic systems. *Rev. Mineral. Geochem.* **69**, 61–120 (2008).

57. S. Mollo, J. D. Blundy, G. Iezzi, P. Scarlato, A. Langone, The partitioning of trace elements between clinopyroxene and trachybasaltic melt during rapid cooling and crystal growth. *Contrib. Mineral. Petrol.* **166**, 1633–1654 (2013).

58. P. E. Wieser, M. Petrelli, J. Lubbers, E. Wieser, S. Özaydin, A. J. R. Kent, C. B. Till, Thermobar: An open-source Python3 tool for thermobarometry and hygrometry. *Volcanica* **5**, 349–384 (2022).

59. Z. Taracsak, M. E. Hartley, R. Burgess, M. Edmonds, F. Iddon, M.-A. Longpré, High fluxes of deep volatiles from ocean island volcanoes: Insights from El Hierro, Canary Islands. *Geochim. Cosmochim. Acta* **258**, 19–36 (2019).

60. M.-A. Longpré, J. Stix, A. Klügel, N. Shimizu, Mantle to surface degassing of carbon- and sulphur-rich alkaline magma at El Hierro, Canary Islands. *Earth Planet. Sci. Lett.* **460**, 268–280 (2017).

61. K. J. Walowski, L. A. Kirstein, J. C. M. De Hoog, T. R. Elliott, I. P. Savov, R. E. Jones, Investigating ocean island mantle source heterogeneity with boron isotopes in melt inclusions. *Earth Planet. Sci. Lett.* **508**, 97–108 (2019).

62. S. Mollo, J. Blundy, P. Scarlato, S. P. de Cristofaro, V. Tecchiato, F. di Stefano, F. Vetere, F. Holtz, O. Bachmann, An integrated *P*-*T*-H_2O-lattice strain model to quantify the role of clinopyroxene fractionation on REE+Y and HFSE patterns of mafic alkaline magmas: Application to eruptions at Mt. Etna. *Earth Sci. Rev.* **185**, 32–56 (2018).

63. C. Jorgenson, O. Higgins, M. Petrelli, F. Bégué, L. Caricchi, A machine learning-based approach to clinopyroxene thermobarometry: Model optimisation and distribution for use in Earth Sciences. *J. Geophys. Res. Solid Earth* **127**, e2021JB022904 (2022).

64. K. Cashman, J. Blundy, Petrological cannibalism: The chemical and textural consequences of incremental magma body growth. *Contrib. Mineral. Petrol.* **166**, 703–729 (2013).

65. J. E. Romero, M. Burton, F. Cáceres, J. Taddeucci, R. Civico, T. Ricci, M. J. Pankhurst, P. A. Hernández, C. Bonadonna, E. W. Llewellin, M. Pistolesi, M. Polacci, C. Solana, L. D'Auria, F. Arzilli, D. Andronico, F. Rodríguez, M. Asensio-Ramos, A. Martín-Lorenzo, C. Hayer, P. Scarlato, N. M. Perez, The initial phase of the 2021 Cumbre Vieja ridge eruption (Canary Islands): Products and dynamics controlling edifice growth and collapse. *J. Volcanol. Geotherm. Res.* **431**, 107642 (2022).

66. J. M. C. Belart, V. Pinel, Pléiades co- and post-eruption survey in Cumbre Vieja volcano, La Palma, Spain. *Dataset on Zenodo*: 10.5281/zenodo.5833771 (2022).

67. R. Civico, T. Ricci, P. Scarlato, J. Taddeucci, D. Andronico, E. del Bello, L. D’Auria, P. A. Hernández, N. M. Pérez, High-resolution Digital Surface Model of the 2021 eruption deposit of Cumbre Vieja volcano, La Palma, Spain. *Sci. Data* **9**, 435 (2022).

68. V. Araña, J. M. Fúster, La erupción del volcán Teneguía, La Palma, Islas Canarias, 1971. *Estud. Geol.* **Teneguía**, 15-18 (1974).

69. V. R. Troll, A. Klügel, M. A. Longpré, S. Burchardt, F. M. Deegan, J. C. Carracedo, S. Wiesmaier, U. Kueppers, B. Dahren, L. S. Blythe, T. H. Hansteen, C. Freda, D. A. Budd, E. M. Jolis, E. Jonsson, F. C. Meade, C. Harris, S. E. Berg, L. Mancini, M. Polacci, K. Pedroza, Floating stones off El Hierro, Canary Islands: Xenoliths of pre-island sedimentary origin in

the early products of the October 2011 eruption. *Solid Earth* **3**, 97–110 (2012).

70. W. F. McDonough, S. S. Sun, The composition of the Earth. *Chem.Geol.***120**, 223–253 (1995).
